# The variety and origin of materials accreted by Bennu’s parent asteroid

**DOI:** 10.1038/s41550-025-02631-6

**Published:** 2025-08-22

**Authors:** J. J. Barnes, A. N. Nguyen, F. A. J. Abernethy, K. Bajo, D. V. Bekaert, E. Bloch, G. A. Brennecka, H. Busemann, J. S. Cowpe, S. A. Crowther, M. Ek, L. J. Fawcett, M. A. Fehr, I. A. Franchi, E. Füri, J. D. Gilmour, M. M. Grady, R. C. Greenwood, P. Haenecour, N. Kawasaki, P. Koefoed, D. Krietsch, L. Le, K. M. Liszewska, C. Maden, J. Malley, Y. Marrocchi, B. Marty, L. A. E. Meyer, T. S. Peretyazhko, L. Piani, J. Render, S. S. Russell, M. Rüfenacht, N. Sakamoto, M. Schönbächler, Q. R. Shollenberger, L. Smith, K. Thomas-Keprta, A. B. Verchovsky, J. Villeneuve, K. Wang, K. C. Welten, J. Wimpenny, E. A. Worsham, H. Yurimoto, L. Zimmermann, X. Zhao, C. M. O’D. Alexander, M. Amini, A. Baczynski, P. Bland, L. E. Borg, R. Burgess, M. W. Caffee, L. C. Chaves, P. L. Clay, J. P. Dworkin, D. I. Foustoukos, D. P. Glavin, V. E. Hamilton, D. Hill, C. H. House, G. R. Huss, T. Ireland, C. E. Jilly, F. Jourdan, L. P. Keller, T. S. Kruijer, V. Lai, T. J. McCoy, K. Nagashima, K. Nishiizumi, R. Ogliore, I. J. Ong, S. M. Reddy, W. D. A. Rickard, S. Sandford, D. W. Saxey, N. Timms, D. Weis, Z. E. Wilbur, T. J. Zega, D. N. DellaGiustina, C. W. V. Wolner, H. C. Connolly, D. S. Lauretta

**Affiliations:** 1 Lunar and Planetary Laboratory, University of Arizona, Tucson, AZ USA; 2 Astromaterials Research and Exploration Science Division, NASA Johnson Space Center, Houston, TX USA; 3 School of Physical Sciences, The Open University, Milton Keynes, UK; 4 Department of Earth and Planetary Sciences, Hokkaido University, Sapporo, Japan; 5 Université de Lorraine, CNRS, CRPG, UMR 7358, Nancy, France; 6 Lawrence Livermore National Laboratory, Livermore, CA USA; 7 Institute of Geochemistry and Petrology, ETH Zurich, Zurich, Switzerland; 8 Department of Earth and Environmental Sciences, The University of Manchester, Manchester, UK; 9 McDonnell Center for the Space Sciences and Department of Earth, Environment, and Planetary Sciences, Washington University in St. Louis, St. Louis, MO USA; 10 Amentum/JETS II Contract, Houston, TX USA; 11 Natural History Museum, London, UK; 12 Barrios/JETS II Contract, Houston, TX USA; 13 Space Sciences Laboratory, University of California, Berkeley, Berkeley, CA USA; 14 Isotope Imaging Laboratory (IIL), Creative Research Institution, Hokkaido University, Sapporo, Japan; 15 Earth and Planets Laboratory, Carnegie Institution for Science, Washington DC, USA; 16 PCIGR, Department of Earth, Ocean and Atmospheric Sciences, The University of British Columbia, Vancouver, British Columbia Canada; 17 Department of Geosciences, Pennsylvania State University, University Park, PA USA; 18 Space Science and Technology Centre, School of Earth and Planetary Sciences, Curtin University, Bentley, Western Australia Australia; 19 Department of Physics and Astronomy and Department of Earth, Atmospheric, and Planetary Sciences, Purdue University, West Lafayette, IN USA; 20 Department of Earth and Environmental Sciences, University of Ottawa, Ottawa, Ontario Canada; 21 Solar System Exploration Division, NASA Goddard Space Flight Center, Greenbelt, MD USA; 22 Solar System Science and Exploration Division, Southwest Research Institute, Boulder, CO USA; 23 Hawaii Institute of Geophysics and Planetary Sciences, School of Ocean and Earth Sciences and Technology, University of Hawaii at Manoa, Honolulu, HI USA; 24 School of the Environment, University of Queensland, St Lucia, Queensland Australia; 25 Stanford Doerr School of Sustainability, Department of Earth and Planetary Sciences, Stanford University, Stanford, CA USA; 26 Department of Mineral Sciences, National Museum of Natural History, Smithsonian Institution, Washington DC, USA; 27 Department of Physics, Washington University in St. Louis, St. Louis, MO USA; 28 John de Laeter Centre, Curtin University, Bentley, Western Australia Australia; 29 NASA Ames Research Center, Moffett Field, CA USA; 30 Department of Geology, Rowan University, Glassboro, NJ USA; 31 Department of Earth and Planetary Science, American Museum of Natural History, New York, NY USA

**Keywords:** Asteroids, comets and Kuiper belt, Meteoritics

## Abstract

The first bodies to form in the Solar System acquired their materials from stars, the presolar molecular cloud and the protoplanetary disk. Asteroids that have not undergone planetary differentiation retain evidence of these primary accreted materials. However, geologic processes such as hydrothermal alteration can dramatically change their bulk mineralogy, isotopic compositions and chemistry. Here we analyse the elemental and isotopic compositions of samples from asteroid Bennu to uncover the sources and types of material accreted by its parent body. We show that some primary accreted materials escaped the extensive aqueous alteration that occurred on the parent asteroid, including presolar grains from ancient stars, organic matter from the outer Solar System or molecular cloud, refractory solids that formed close to the Sun, and dust enriched in neutron-rich Ti isotopes. We find Bennu to be richer in isotopically anomalous organic matter, anhydrous silicates, and light isotopes of K and Zn than its closest compositional counterparts, asteroid Ryugu and Ivuna-type (CI) carbonaceous chondrite meteorites. We propose that the parent bodies of Bennu, Ryugu and CI chondrites formed from a common but spatially and/or temporally heterogeneous reservoir of materials in the outer protoplanetary disk.

## Abstract (web summary)

Bennu comprises components of intra- and extra-Solar System origins. The parent bodies of Bennu, Ryugu and CI chondrites likely formed from a shared but heterogeneous reservoir in the outer parts of the solar protoplanetary disk.

## Main

NASA’s Origins, Spectral Interpretation, Resource Identification, and Security-Regolith Explorer (OSIRIS-REx) mission surveyed (101955) Bennu from 2018 to 2021 and delivered 121.6 g of its regolith (unconsolidated granular material) to Earth on 24 September 2023^1,2^. Bennu is an ~500-m-diameter near-Earth asteroid. It is a rubble pile, consisting of reaccumulated fragments of a much larger parent body (≥100 km) that was collisionally disrupted in the main asteroid belt^3^. Unlike meteorites, the pristine Bennu samples returned by OSIRIS-REx have not been subjected to heating from entry through Earth’s atmosphere and have experienced minimal or no interaction with the ambient atmosphere and biosphere. These qualities make them ideal for probing the nature and formation of early planetesimals, particularly their volatile and organic contents.

Remote sensing by OSIRIS-REx^4–6^ combined with the first laboratory analyses of the regolith samples^2^ showed that Bennu’s surface material is composed of hydrated clay minerals (phyllosilicates), magnetite, sulfides, carbonates, organic matter, phosphates, and small abundances of anhydrous silicates and oxides including olivine, pyroxene and spinel. These findings established that Bennu’s parent body experienced extensive mineralogical changes, whereby most of the original dust inherited from the protoplanetary disk, including metals and anhydrous and amorphous silicates^7^, was aqueously altered to secondary phases. This alteration was likely caused when water, carbon dioxide, ammonia^8^ and other ices accreted by the parent body melted due to heat generated from the decay of short-lived radioactive nuclides and impact events.

Detailed study of the returned samples is required to understand the diversity of materials accreted by the parent asteroid, the chemical and isotopic reservoirs in the protoplanetary disk where it formed, and the extent to which it was hydrothermally altered. We investigated the bulk elemental and isotopic composition of Bennu aggregate material—loose, unsorted particles <0.5 cm—and the in situ isotopic compositions of individual components, including presolar grains, organic matter and anhydrous silicates. Comparing the composition of Bennu samples with those of carbonaceous chondrites (CCs) and samples of asteroid (162173) Ryugu returned by the Japan Aerospace Exploration Agency’s Hayabusa2 mission^9,10^ places the accretion history and chemical evolution of Bennu’s parent body in the broader context of other primitive astromaterials.

## Results

### Bulk chemical and isotopic compositions

**Extended Data Fig. 1:**
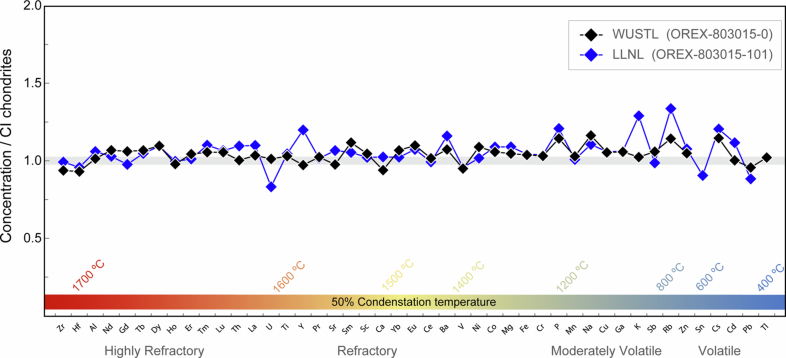
Bulk chemical composition of two Bennu samples (OREX-803015-0 and OREX-803015-101). The relative abundances of 44 elements are shown normalized to those of CI chondrites (gray bar;^134^). The abundances are mostly comparable between these two samples measured at different laboratories. OREX-803015-0 data are from Lauretta & Connolly et al.^2^. Errors are not shown. As discussed in the Methods, most concentrations are within 10% of reference values. Source data

The bulk abundances of 44 elements in Bennu samples were analysed by inductively coupled plasma mass spectrometry (ICP-MS) (Methods and Supplementary Tables 1 and 2). The Bennu material has a solar-like refractory element composition mostly within 5% of solar values^11^. We observed depletions in uranium (U), tin (Sn) and lead (Pb), alongside enrichments in fluid-mobile elements including yttrium (Y), barium (Ba), phosphorus (P), sodium (Na) and potassium (K) (Extended Data Fig. 1), generally consistent with previous results^2^.

**Extended Data Fig. 2:**
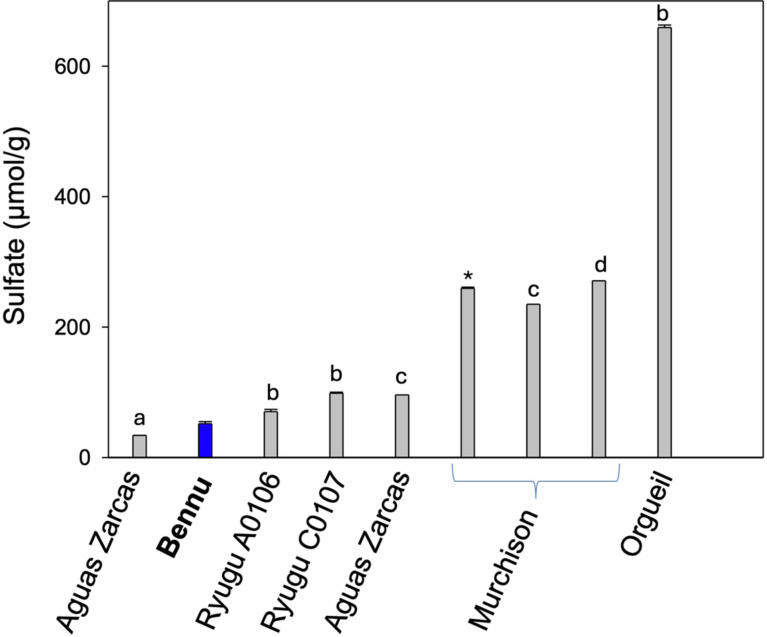
Sulfate composition of Bennu (OREX-803001-111) compared to other carbonaceous astromaterials measured in water extracts. Bennu and Murchison (*) data are from this study. Other data sources: a-Aguas Zarcas^99^; b-Ryugu and Orgueil^100^; c- data on Aguas Zarcas and Murchison water washes from unhydrolyzed hot-water extract, run in the same lab with the same ion chromatography method as for Bennu; d-Murchison^98^ (see Supplementary Table 3). Source data

The abundances of soluble anions were determined using ion chromatography (Methods, Extended Data Fig. 2 and Supplementary Table 3). Of the suite analysed, we detected inorganic sulfate (SO_4_^2−^, 51.77 ± 3.11 µmol g^−1^) and phosphate (PO_4_^3−^, 0.08 ± 0.01 µmol g^−1^). These results are consistent with previous studies^2,12^ indicating the presence of water-soluble sulfate and phosphate-bearing minerals in Bennu samples.

**Extended Data Fig. 3:**
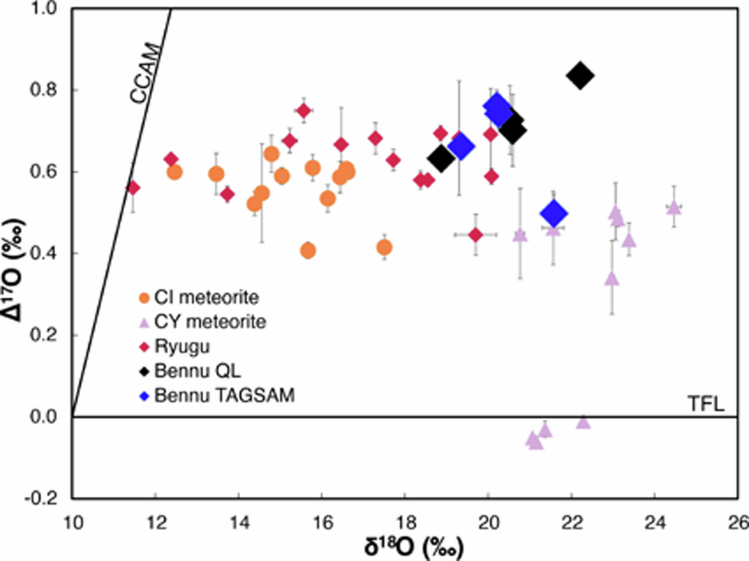
Bulk oxygen isotopic compositions of Bennu samples compared with other astromaterials. Oxygen isotopic composition of samples retrieved from the avionics deck of the sample return canister (“QL”) (OREX-501066-0, OREX-501067-0, OREX-501047-0, OREX-501042-0)^2^ and from within the sample collector (“TAGSAM”) (OREX-803110-0, OREX-803136-0, OREX-803137-0, OREX-803140-0) (this study, Methods). Ryugu and meteorite data are from Greenwood et al.^26^ and references therein. The TFL and CCAM are described in the Methods. Error bars represent 2SE measurement errors and for Bennu samples they are smaller than data points where not visible. Source data

The weighted average of four laser-assisted fluorination analyses of Bennu samples yields a bulk oxygen (O) isotopic composition of +11.2 ± 0.8‰ for δ^17^O, +20.2 ± 1.8‰ for δ^18^O and +0.66 ± 0.24‰ for Δ^17^O (2 standard errors (s.e.); Methods and Extended Data Fig. 3), consistent with the weighted average composition for Bennu samples exposed to air^2^ (Supplementary Table 4). The δ notation indicates parts per thousand deviations from a standard composition. The Δ^17^O value is used to describe the mass-independent deviation from the terrestrial mass fractionation line (or slope of 0.52) on an oxygen three-isotope plot. The variation shown by the samples exceeds typical analytical precision by at least an order of magnitude at the 2*σ* level (Methods). The ranges of δ^17^O and δ^18^O in these samples are less than that reported previously^2^. The most extreme isotopic compositions are represented in the fine and intermediate-sized particles retrieved from the avionics deck^2^, which may indicate varying abundances of distinct O isotopes across different particle sizes.

**Extended Data Fig. 4:**
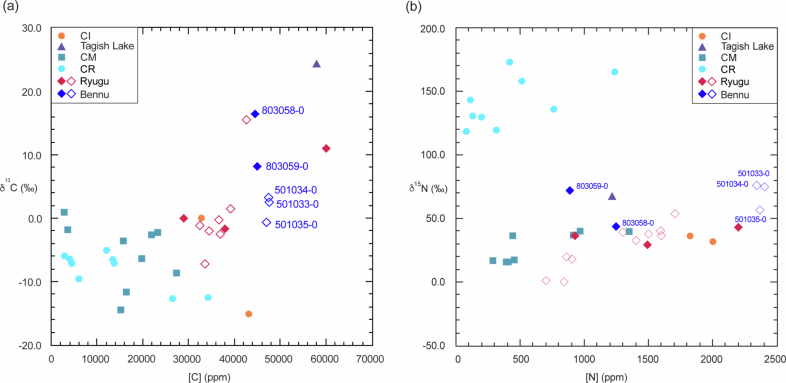
Abundances and isotopic composition of C and N in Bennu samples (OREX-803058-0 and OREX-803059-0) compared to other carbonaceous astromaterials. Summed abundance and isotopic composition of (**a**) carbon and (**b**) nitrogen obtained by stepped combustion of Bennu samples compared to carbon-rich carbonaceous chondrites. All data are from the OU laboratory, apart from Ryugu (open red diamonds^18,109,110^) and other Bennu data (open blue diamonds^2^) (Supplementary Table 5). Error bars on isotopic composition represent 2 SD and are smaller than the symbol. Source data

**Extended Data Fig. 5:**
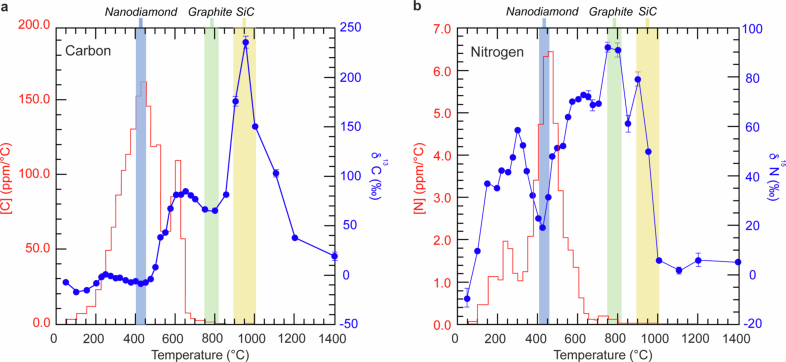
Bulk C and N abundances and isotopic compositions of Bennu sample (OREX-803058-0). Stepped combustion profiles from Bennu sample OREX-803058-0 (Methods) for (**a**) carbon and (**b**) nitrogen. Abundances are plotted as the red histogram, scaled on the left axis; isotopic compositions are plotted as blue dots, scaled on the right axis. The colored bars indicate the peak combustion temperature for nanodiamonds (blue)^135^, and likely graphite (green) and silicon carbide (yellow), although the release temperature is likely broader, primarily as a function of crystallinity and grain size^136^. The C isotopic compositions of approximately –10 ‰ and +70 ‰ indicate volatile organics and carbonates, respectively. For N, volatile organics coincide with δ^15^N ~ + 20 ‰ and less volatile organics from ~+40 to +100 ‰. Error bars represent 2 SD and are smaller than the symbol where not visible.

Using stepped-combustion isotope ratio mass spectrometry, we obtained total carbon (C) contents of 4.42 wt% and 4.45 wt% and nitrogen (N) contents of 882 ppm and 1,246 ppm in 2 samples (Methods and Supplementary Table 5). The corresponding weighted summed values for δ^13^C are +16.7‰ and +8.3‰, and for δ^15^N are +43.8‰ and +72.2‰ (Extended Data Fig. 4). The C contents are similar to those reported in other Bennu samples^2,8^, but the N contents are lower (Extended Data Fig. 4). Our data overlap the δ^15^N values reported earlier^8^ and show higher δ^13^C values, which may result from greater contribution of carbonates or presolar grains in the small masses analysed here (<2 mg; Methods). Distinct groupings in the C data indicate the presence of three C-bearing components: organics (δ^13^C ≤ −10‰), carbonates (for example, Fe,Mg-carbonate; δ^13^C > +43‰) and presolar grains (diamonds, graphite and silicon carbide (SiC); Extended Data Fig. 5). The N data indicate at least three components: volatile organics (δ^15^N ≈ +20‰), less volatile organics (δ^15^N ≈ +40–100‰) and presolar grains (Extended Data Fig. 5).

**Fig. 1:**
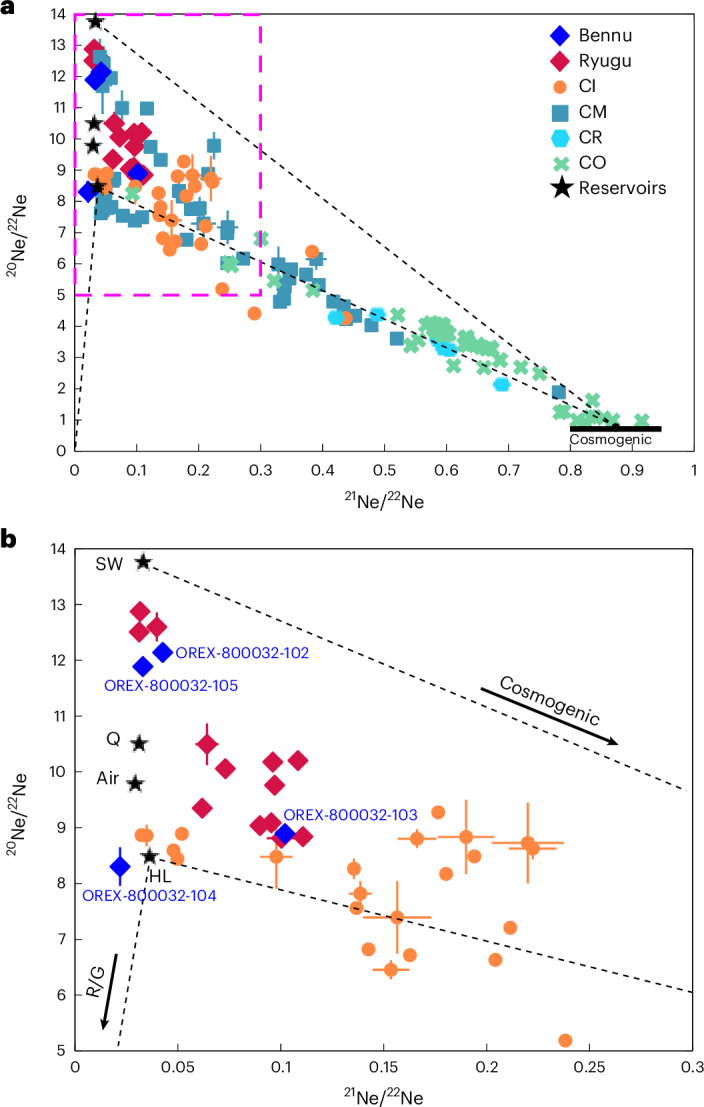
Bulk Ne isotopic composition of Bennu samples (OREX-800032-102, -103, -104 and -105) compared with Ryugu and CCs. **a**,**b**, Neon three-isotope plot (**a**) and restricted isotope ratio range plot (**b**). The Ne isotopic composition of a sample represents mixing between solar wind (SW), cosmogenic, phase Q (Q) and presolar (HL, R/G) components of Ne in varying proportions, as well as terrestrial air. The black dashed lines represent the mixing lines between these components. The pink dashed box in **a** denotes the bounds of **b**. CCs are abbreviated as follows: CI, Ivuna type; CM, Mighei type; CR, Renazzo type; CO, Ornans like. See Methods for sources of non-Bennu data. Error bars represent 1*σ* measurement uncertainties. Source data

**Extended Data Fig. 6:**
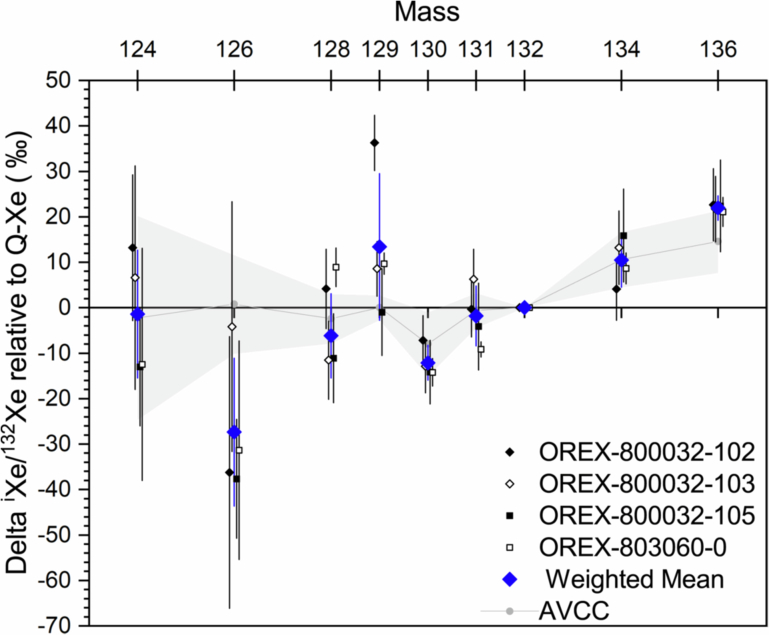
Bulk Xe isotopic composition of Bennu particles (OREX-800032-102, OREX-800032-103, OREX-800032-105, OREX-803060-0). Xe isotopic compositions are presented relative to Q-Xe, using ^132^Xe as the reference isotope. AVCC, average carbonaceous chondrite^13,137^. Error bars represent 2-sigma. Source data

Noble gas analyses indicate high abundances of argon-36 at 167 × 10^−8^ cm^3^ STP g^−1^ to 211 × 10^−8^ cm^3^ STP g^−1^ (where STP is standard temperature and pressure) (Methods and Supplementary Tables 6–8). In triple-neon-isotope space (Fig. 1), Bennu materials show a spread in neon (Ne) isotopic compositions reflecting contributions from (1) trapped noble gases, including Ne from phase Q, the major carrier of planetary noble gases in CCs, which is likely associated with organic matter and C-rich presolar grains^13^; (2) solar wind implanted into surface materials; and (3) cosmogenic Ne produced through galactic and solar cosmic rays. We find xenon-132 concentrations of ~1.8 × 10^−8^ cm^3^ STP g^−1^ to 2.6 × 10^−8^ cm^3^ STP g^−1^. The Xe isotope compositions are consistent with the average CC composition, that is, phase Q plus slight enrichments in heavy and light isotopes (‘Xe-HL’) from presolar nanodiamonds^13,14^ (Extended Data Fig. 6). We also find excesses in radiogenic ^129^Xe from the decay of ^129^I.

**Fig. 2:**
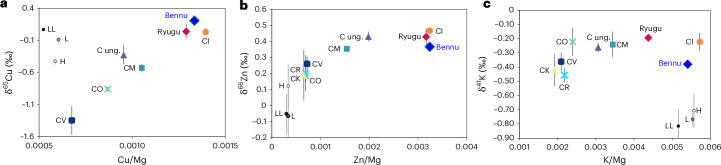
Elemental abundance ratios versus isotopic composition of moderately volatile elements in Bennu sample OREX-803015-0 compared with Ryugu and CCs. **a**–**c**, Bulk copper (Cu) (**a**), zinc (Zn) (**b**) and potassium (K) (**c**) elemental abundances compared to their isotopic compositions with respect to Mg. The inverse Mg-normalized values are used to compensate for the variable (1) metal-silicate fractionation, (2) refractory inclusion abundances and (3) extent of alteration (H_2_O content) across the different samples. CK, Karoonda-type; CV, Vigarano-type; C ung., ungrouped CCs. Ordinary chondrites include H, L and LL types. See Methods for sources of non-Bennu data. Data are presented as mean values with 2 s.e. error bars. Source data

**Fig. 3:**
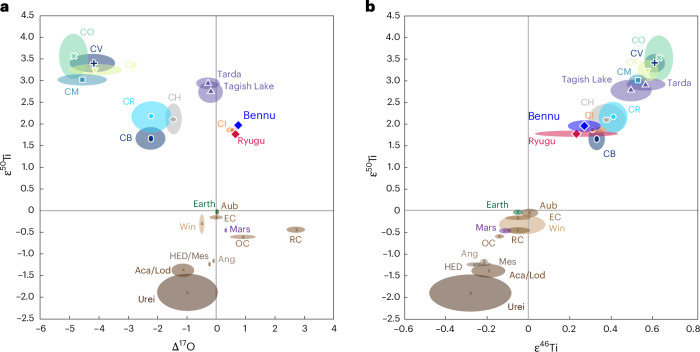
Bulk titanium and oxygen isotopic compositions of Bennu samples (OREX-803015-100 and OREX-803015-101) in relation to other astromaterials. **a**, Bulk ɛ^50^Ti versus oxygen isotopic composition. **b**, Bulk ɛ^50^Ti versus ɛ^46^Ti isotopic composition. CB, Bencubbin-like CCs; OC, ordinary chondrites; RC, Rumuruti chondrites; EC, enstatite chondrites; Aub, aubrites; Win, winonaites; Ang, angrites; HED/Mes, howardite–eucrite–diogenite and mesosiderite; Aca/Lod, acapulcoite and lodranite; Urei, ureilite. See Methods for sources of non-Bennu data. The symbols at the centre of ovals represent the centre of the range of values. The sizes of the ovals represent the range of data for each material, including reported 2 s.d. uncertainty on measurements. Source data

The Bennu samples show mass-dependent isotope compositions (where deviation in isotope abundances scales with the mass of the isotopes involved) of K, copper (Cu) and zinc (Zn): δ^41^K of −0.38 ± 0.03‰, δ^65^Cu of +0.21 ± 0.02‰ and δ^66^Zn of +0.37 ± 0.02‰ (2 s.e.; Fig. 2), as measured by multi-collector ICP-MS (Methods and Supplementary Table 9). The non-mass-dependent (nucleosynthetic) titanium (Ti) isotopic composition of the Bennu samples averages +0.27 ± 0.08 ε^46^Ti, −0.02 ± 0.05 ε^48^Ti and +1.98 ± 0.08 ε^50^Ti (2 s.e.; Fig. 3), where the ε notation signifies parts per ten thousand deviations relative to a terrestrial standard (Methods and Supplementary Table 10).

### In situ isotopic compositions

**Fig. 4:**
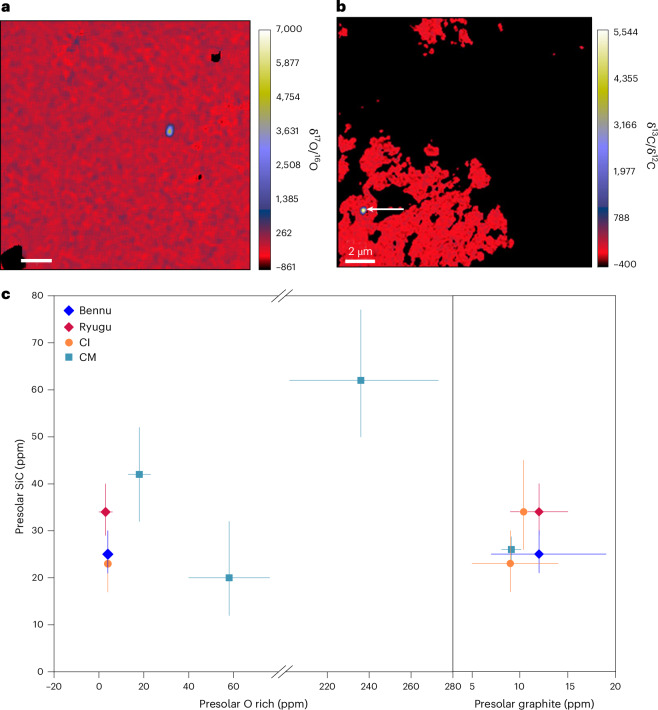
Isotopic mapping to identify presolar grains in Bennu samples (OREX-501018-100, OREX-501049-0 and OREX-501080-0) and comparison of their abundances with other carbonaceous astromaterials. **a**, NanoSIMS δ^17^O/^16^O ratio image of a region containing an isotopically anomalous O-rich presolar grain. Scale bar, 2 µm. **b**, NanoSIMS δ^13^C/^12^C ratio image of a region containing a presolar SiC grain that is denoted by the arrow. **c**, Abundances of presolar SiC, O-rich grains and graphite in Bennu (this study) compared with Ryugu, CI and CM chondrites (see Methods for sources of non-Bennu data). The presolar O-rich abundance for CI chondrites is an upper limit. Error bars are 1 s.d. around mean values. Source data

**Extended Data Fig. 7:**
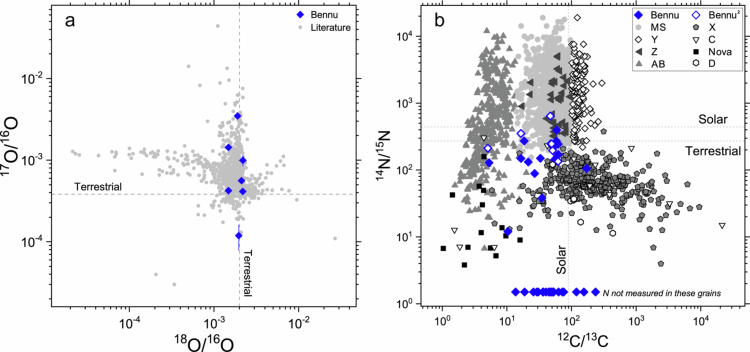
Isotopic compositions of presolar grains in Bennu samples which reflect their stellar origins. (**a**) O isotopic compositions of presolar silicates and oxides and (**b**) C and N isotopic compositions of Bennu presolar SiC (this study and^2^) compared to the literature^138,139^. Error bars (1-sigma) are only shown for the O-rich presolar grains in Bennu for clarity. Errors can be found in Supplementary Table 11. Source data

**Extended Data Fig. 8:**
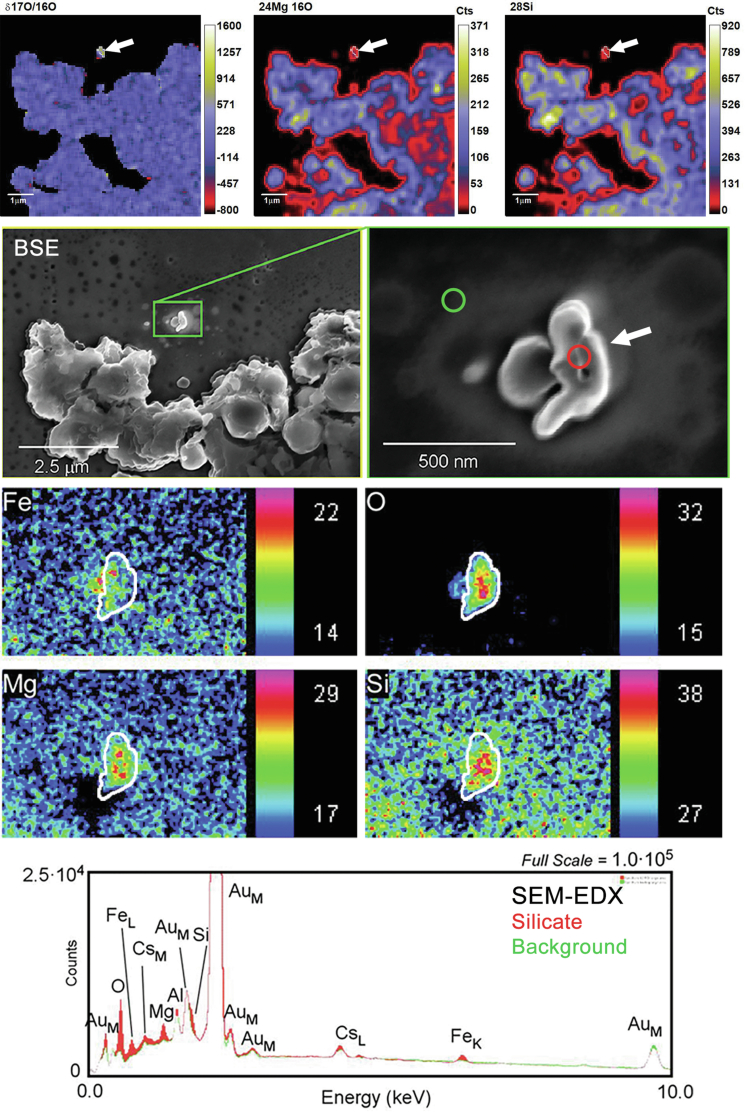
NanoSIMS and SEM-EDX characterization of a presolar silicate grain from Bennu sample OREX-501018-100. NanoSIMS ratio and ion images (top images) of a region containing a ^17^O-rich presolar silicate grain (arrows). The elemental maps and spectra from SEM-EDX analysis confirm the grain is a ferromagnesian silicate.

Presolar grains are identified by their highly anomalous isotopic compositions due to nucleosynthetic reactions that occurred in their parent stars (for example, ref. ^15^). We searched for preserved, individual presolar grains by in situ C, N, O and silicon (Si) isotopic mapping of the phyllosilicate-rich matrix material using nanoscale secondary ion mass spectrometry (NanoSIMS; Methods and Supplementary Tables 11 and 12). On the basis of the highly anomalous O-isotope ratios (δ^17^O −689‰ to +8,067‰ and δ^18^O −262‰ to +98‰; Extended Data Fig. 7), 7 O-rich presolar grains were identified, including 2 silicates. The chemical compositions of two O-rich presolar grains, determined by scanning electron microscopy–energy dispersive X-ray spectroscopy (SEM-EDS), indicated that one is a ferromagnesian silicate (Extended Data Fig. 8) and one is an aluminium (Al) and magnesium (Mg)-bearing oxide. In addition, 39 presolar SiC and 6 presolar graphite grains were identified with anomalous C and/or N isotopic compositions (δ^13^C −737‰ to +15,832‰ and δ^15^N −310‰ to +21,661‰). The abundances of presolar SiC, graphite and O-rich grains are ${25}_{-4}^{+5}\,{\mathrm{ppm}}$, ${12}_{-5}^{+7}\,{\mathrm{ppm}}$ and 4 ± 2 ppm, respectively (Fig. 4).

**Extended Data Fig. 9:**
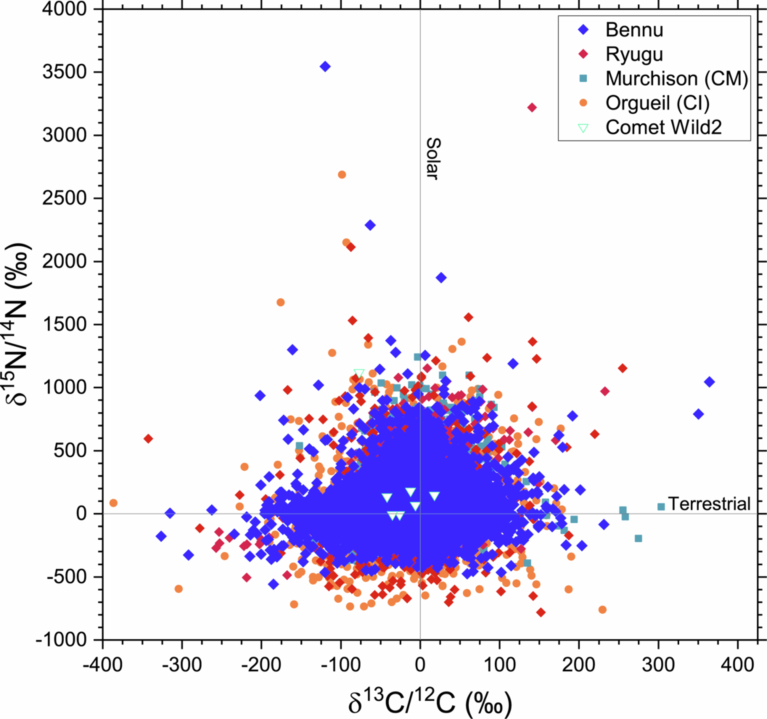
C and N isotopic compositions of organic matter in Bennu sample OREX-501018-100. C and N isotopic compositions determined by in situ NanoSIMS isotope mapping show discrete regions of organic matter having highly anomalous N isotopic compositions and a smaller range of C isotopic compositions. Sources of non-Bennu data are: Ryugu^29,30^; Orgueil^29^; Murchison acid-insoluble residue^28^; comet Wild2^32,140^. Errors are not shown for clarity and can be found in Supplementary Table 13. The average error for the Bennu data is 85 ‰ for δ^15^N, and 42 ‰ for δ^13^C. Source data

NanoSIMS mapping showed that organic matter in Bennu samples occurs as discrete phases, including nanoglobules, and in a diffuse form throughout the matrix^2^ (Methods and Supplementary Table 13). Discrete regions of organic matter had δ^15^N values from −558‰ to +3,545‰, δ^13^C values from −326‰ to +364‰ and δD values from −920‰ to +11,413‰ (Extended Data Fig. 9). Organic matter having anomalous isotopic compositions in H, N and C relative to the bulk compositions comprised 1.1, 0.6 and 0.04 area%, respectively, of the total area of material analysed (Methods).

**Fig. 5:**
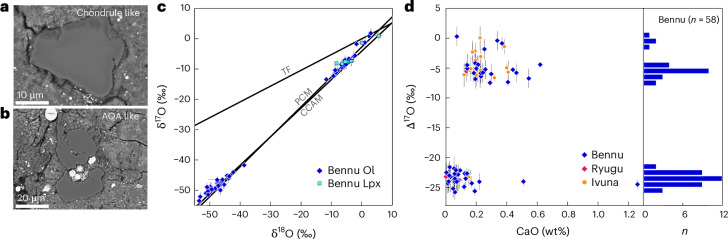
Petrography and oxygen isotopic and chemical compositions of anhydrous silicate minerals in Bennu samples (OREX-501054-0, OREX-501059-0, OREX-803114-0, OREX-800045-103 and OREX-800045-107). **a**,**b**, Backscattered electron images of a chondrule-like olivine grain (Δ^17^O = −7‰; **a**) and an AOA-like olivine grain (Δ^17^O = −23‰; **b**). **c**, Oxygen isotopic compositions of individual olivine (Ol) and low-Ca pyroxene (Lpx). Oxygen isotopic compositions reflect three different groupings: a solar-like composition as found in primitive components of other CCs (CAIs and AOAs), a ^16^O-enhanced composition at Δ^17^O = −5‰ and a near-terrestrial (planetary) composition. TF, terrestrial fractionation line; CCAM, carbonaceous chondrite anhydrous mineral line; PCM, primitive chondrule mineral line (Methods). **d**, CaO contents (wt%) versus oxygen isotopic compositions (Δ^17^O) of olivine grains in Bennu (this study), Ryugu and the Ivuna CI chondrite^33^. The right panel is a histogram of Δ^17^O values in Bennu olivine grains. Error bars in **c** and **d** are 2 s.d. measurement errors. Source data

We determined the O isotopic compositions of refractory silicate minerals—specifically, olivine and low-calcium pyroxene—in situ by SIMS and NanoSIMS (Methods and Supplementary Table 14). These minerals show mass-independent fractionation of O isotopes and a range of compositions, from ^16^O-rich grains with near-solar (δ^17^O, δ^18^O < −40‰) compositions to ^16^O-poor grains with near-planetary (δ^17^O, δ^18^O ≈ 0‰) isotopic compositions (Fig. 5).

## Discussion

### Bennu’s bulk composition compared with other primitive samples

Bennu samples strongly resemble Ivuna-type (CI) chondrites, with broadly similar bulk chemical compositions (Extended Data Fig. 1). The bulk compositions of CCs reflect the origins and alteration histories of their parent bodies, with CI chondrites most closely resembling the solar photosphere^11^. Hence, they are considered the most chemically primitive. However, Bennu, like Ryugu, is enriched in P compared with CI chondrites^2^. The abundant P and presence of sulfate and phosphate ions in Bennu (Extended Data Figs. 1 and 2) indicate contributions from organics and evaporite minerals such as soluble salts and phosphates^2,8,12^. The relatively low abundance of sulfate suggests that the conditions during alteration promoted sulfate loss, such as fluid flow through late-stage open systems or reducing environments.

We identified the same types of C- and N-rich components—presolar grains, organics and carbonates—as those found in Ryugu, CI and Mighei-type (CM) chondrites^16^ (Extended Data Fig. 5). However, we find that Bennu, like Ryugu, is more C-rich than CCs (Extended Data Fig. 4). The samples show a range in bulk N abundance, overlapping abundances in CCs and Ryugu. Isotopically, the samples analysed here show δ^13^C values similar to some Ryugu particles and more elevated than CIs and CMs, whereas the δ^15^N values are consistent with those samples.

Several isotopic systems imply that Bennu’s parent body, like Ryugu’s, retained a primary volatile inventory, consistent with formation and preservation in a relatively cold, unprocessed region of the early solar nebula. The Bennu samples show similar noble gas abundances to Ryugu samples and heterogeneity in Ne and Xe isotopes comparable to other primitive CCs and Ryugu^17–19^ (Fig. 1 and Extended Data Fig. 6). Endmember compositions of trapped noble gases (those not implanted by solar wind) in Bennu samples are consistent with those of other aqueously altered materials, including CI, CM and Renazzo-type (CR) chondrites, indicating contributions of noble gases from Q-bearing phases and presolar grains^17,20^. The moderately volatile element (MVE) isotope systems (K, Cu and Zn) closely resemble those of CIs and Ryugu^21,22^. Bennu’s K and Zn isotopic compositions are slightly enriched in lighter isotopes^21–23^ (Fig. 2) suggesting minimal volatile loss and limited thermal processing.

Small variations in isotopic abundances of transition metals (for example, Ti, Cr, Mo) in astromaterials arose because of heterogeneous distribution and incomplete mixing of presolar dust, the carriers of these nucleosynthetic signatures, in the early Solar System^24^. The neutron-rich Ti isotope signatures indicate that Bennu shares a nucleosynthetic heritage with other CCs and is most similar to CIs and Ryugu^10^ (Fig. 3). The Δ^17^O values also indicate similar formation environments. The δ^18^O values of the CIs^25,26^, however, are markedly lower than Bennu’s (Extended Data Fig. 3), likely reflecting modification of CIs by exposure to Earth’s atmosphere and weathering.

Altogether, the bulk characteristics of Bennu indicate that it is chemically primitive and has close chemical and isotopic affinity to Ryugu and CIs.

### Origins of the parent body’s primary accreted components

The oldest primary constituents in Bennu samples, like in other primitive astromaterials, are submicrometre-sized presolar grains with isotopic compositions indicating diverse stellar sources (Extended Data Fig. 7). Most of the Bennu SiC grains have C and N isotopic compositions that are consistent with nucleosynthetic reactions occurring in low-mass asymptotic giant branch (AGB) stars. Grains with large ^15^N enrichments likely have nova or supernova origins. Type AB grains have ^12^C/^13^C ratios <13.5 and could have come from J-type C stars, born-again AGB stars or supernovae^15^. The graphite grains originate from AGB stars or supernova. The O-rich presolar grains include ^17^O-rich grains of AGB star or supernova origins and ^17^O-poor grains of supernova origin.

Organic matter that is isotopically indistinguishable from the bulk composition may have formed in the parent body or in the nebula. A fraction (<10%) of organic matter in carbonaceous astromaterials, including Ryugu, has large isotopic anomalies in H, C and N that are postulated to result from low-temperature (~10–40 K) chemical reactions in the molecular cloud or outer protoplanetary disk^27–29^. We found the ranges of H, C and N isotopic compositions of insoluble organic matter in Bennu to be similar to those in CIs and CMs^28,29^, Ryugu^29–31^, and comet Wild 2 samples returned by NASA’s Stardust mission^32^ (Extended Data Fig. 9). These compositional and isotopic parallels between bulk and in situ data indicate that Bennu, like Ryugu, preserves a diverse suite of primitive organic and volatile-rich materials.

**Extended Data Fig. 10:**
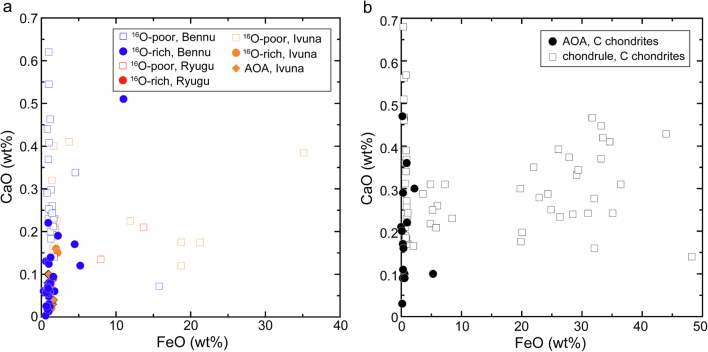
Relationships between CaO and FeO contents for 16O-rich and 16O-poor olivine grains in Bennu (OREX-803114-0) and other carbonaceous astromaterials. (**a**) CaO versus FeO content of olivine and pyroxene in Bennu (this study), Ryugu and CI chondrite Ivuna^33^. (**b**) CaO versus FeO content of olivine and pyroxene in AOAs and chondrules in CCs^141–144^. Source data

Mineral assemblages that formed close to the Sun include refractory inclusions (amoeboid olivine aggregates (AOAs) and calcium–aluminium-rich inclusions (CAIs)) and chondrules consisting of anhydrous Mg,Fe-rich silicates and oxide minerals. Their O isotopic compositions reflect the solar nebula composition (^16^O rich) and subsequent isotopic exchange with a ^16^O-poor reservoir. They are common in most types of CC, but are rare in CIs, Ryugu and comet Wild 2^33–36^. The Bennu samples have minor abundances of submillimetre anhydrous silicates and oxides including olivine, pyroxene and spinel^2^. The anhydrous silicate grains in the Bennu samples we analysed have strong chemical (CaO and FeO content; Extended Data Fig. 10) and isotopic affinity to ^16^O-rich AOAs and ^16^O-poor chondrules found in CCs (Fig. 5), suggesting that they are fragments of these inclusions. Thus, these minerals represent some of the earliest Solar System condensates that accreted into Bennu’s parent body. The similar bulk Ti isotopic compositions of Bennu, Ryugu and CIs^24,37^ (Fig. 3) suggest similar, although not identical, proportions of AOAs, chondrules, CAIs and matrix. This supports the interpretation from petrologic characterization of Bennu samples that the parent body formed predominantly from a mixture dominated by dust, ices and organics, with minor contributions of AOAs, chondrule and CAI-like solids^7^.

Our in situ observations demonstrate that the materials accreted by Bennu’s parent asteroid had diverse origins, and some survived subsequent processing.

### Geological activity within Bennu’s parent body

Presolar C-rich grains can be altered or destroyed by thermal metamorphism and prolonged oxidation^38^. The abundances of C-rich presolar grains in Bennu samples (25 ppm SiC and 12 ppm graphite) are comparable to those in unheated carbonaceous astromaterials, including CIs and Ryugu^30,38,39^ (Fig. 4). Preservation of these presolar grains indicates that Bennu’s parent body did not experience prolonged thermal metamorphism exceeding ~400 °C (ref. ^38^), in agreement with the much lower temperatures of aqueous alteration inferred from evaporite mineralogy (<50 °C (refs. ^7,12^)).

Bennu’s unfractionated bulk chemistry suggests closed-system aqueous alteration. However, enrichments in some fluid-mobile elements^2^ (Extended Data Fig. 1) are consistent with an open system. These enrichments, along with detected phosphate ions, suggests the addition of chemically distinct fluid(s)^2,8,12^.

Presolar silicates are rapidly altered by hydration, and thus their abundances are sensitive tracers of aqueous activity^30,40^. The least aqueously altered CCs, petrographically classified as types 2 and 3, have abundances up to ~250 ppm (ref. ^40^), whereas no presolar silicates have been identified in the most aqueously altered type 1 CIs^39^. That Bennu and Ryugu preserve presolar O-rich grains, albeit at similarly low abundances (4 ± 2 ppm and 3 ± 2 ppm, respectively)^30,39^, suggests that their parent bodies experienced an intermediate degree of alteration between those of type 1 and type 2/3 meteorites.

Similarly, the nebular anhydrous silicates in Bennu indicate that aqueous alteration, although extensive, was not complete (that is, not all anhydrous silicates converted to hydrated silicates). The abundance of anhydrous silicates (1–4 vol%)^2,7^ is higher than that within the major hydrated lithology of Ryugu (<0.1 vol%) but is comparable to a less-altered Ryugu clast (3.9 vol%)^41^. This may indicate that the Bennu samples experienced less alteration than the Ryugu samples. However, their similar presolar silicate abundances suggest similar degrees of alteration; therefore, an alternative explanation could be that Bennu’s parent body started with a greater proportion of anhydrous Solar System silicates than Ryugu’s.

The H isotopic composition of organics in Bennu samples provides key constraints on the extent of aqueous alteration. Bulk δD values of insoluble organic residues in CCs have been shown to decrease with increasing aqueous alteration, while δ^13^C and δ^15^N values remain largely unaffected^42^. Similarly, the destruction of D-enriched domains in organics has been linked to hydrothermal processing^28^. The preservation of pronounced D enrichments in Bennu organic matter and the high abundance of organics exhibiting H isotopic anomalies supports the interpretation that hydration was incomplete. The Bennu samples contain more than two times the abundance of isotopically anomalous organic matter than samples of the hydrated Ryugu lithology^29,30,41^ and Orgueil^39^. The distribution and abundance of amino acids^8^ also suggest that the parent body was less aqueously altered than type 1 chondrites and Ryugu.

We find a similar removal of the argon (Ar)-rich component carrier(s), which are rapidly altered by hydration, as in the most aqueously altered CMs and CIs^20^. This contrasts with the observations of presolar and anhydrous silicates and organic matter in Bennu that suggest a lower degree of aqueous alteration than CIs. The Ar-rich component may therefore be more sensitive to aqueous alteration than silicates.

The isotopically light MVE composition of Bennu samples analysed here, relative to the CIs’ average composition, could indicate that the parent bodies started off with distinct MVE compositions. Alternatively, these data may reflect limited sampling of the full range of Bennu’s K and Zn isotopic compositions resulting from aqueous alteration. We favour the latter because K and Zn are fluid mobile, and it has been shown that aqueous alteration could explain the range of K and Zn isotopic compositions among CI-like materials (for example, ref. ^43^).

Our findings place Bennu in an intermediate position along the CC alteration continuum, bridging the heavily altered type 1 and the less-altered type 2/3 astromaterials, and recording the complex interplay of primordial accretion, aqueous activity and organic chemistry in early Solar System bodies. Crucially, the higher abundance of anhydrous silicates and isotopically anomalous organic matter in Bennu compared with Ryugu samples suggests that their respective parent bodies accreted different mixtures of these materials. It is also possible that the aggregate samples analysed in this study do not represent the full range of aqueous alteration experienced by Bennu’s parent body. The lithologies and their proportions in the aggregate samples are not yet constrained^2^.

### The reservoir from which Bennu’s parent body formed

Given the data presented here, particularly the nucleosynthetic signatures, abundances of C and N, and high abundances of anhydrous silicates and isotopically anomalous organic matter, we conclude that Bennu’s parent body formed in a region containing presolar SiC, graphite, oxides and silicates, as well as organics and ices^8^ from the outer Solar System and interstellar medium. This region also contained refractory silicate minerals that were likely transported from hot, inner regions of the protoplanetary disk to colder areas where ice was stable.

Our data reinforce existing dynamical and geologic evidence for common histories of the parent bodies of Bennu and Ryugu^3,41^. The bulk solar elemental abundances in samples from both asteroids affirms their primitive nature (Extended Data Fig. 1). Their shared mineral inventories^2,12,41^ indicate that both underwent hydrothermal alteration by alkaline, salt-rich water, before catastrophic disruption and subsequent reaccumulation into rubble-pile asteroids^3,41^.

Two isotopically distinct reservoirs in the Solar System are well resolved, representing non-carbonaceous and carbonaceous astromaterials^24,44^. This isotopic divide indicates an early spatial separation within the protoplanetary disk and a dynamical barrier that prevented large-scale mixing. Candidate mechanisms include the early formation of Jupiter^45^, a pressure maximum within the protoplanetary disk^46^, possibly related to the heliocentric distance where water ice condensed (known as the ‘snowline’)^47^, or a combination thereof. Some studies suggest the presence of substructures or subreservoirs within at least the inner disk^37^, and possibly a third reservoir farther out in the outer Solar System corresponding to the CI-, Ryugu and Bennu materials^48^. The neutron-rich Ti isotope signatures measured here suggest that the reservoir(s) sourcing the parent bodies of Bennu, Ryugu and CIs were distinct from those of all other chondritic meteorites. Moreover, the overlapping ranges of O isotopes in Bennu and Ryugu samples^9,26^ (Extended Data Fig. 3) implies a common primordial source or exposure to similar physicochemical environments during early Solar System evolution.

Bennu’s parent asteroid could have accreted in a reservoir located close to the water snowline that was seeded with sunwards-drifting ice, refractory solids and dust^47^. However, the CIs likely derive from parent bodies that accreted at distances >5 au (refs. ^41,49^). Moreover, exogenous clasts in Ryugu samples may have originated beyond the trans-Neptunian region^30^. The data support an outer Solar System location, possibly beyond the orbit of Saturn, for formation of Bennu’s parent asteroid, particularly the high abundance of organic matter with H and N isotope anomalies reported here and the elevated ammonia content and ^15^N enrichments in the soluble organics reported previously^8^. These characteristics are shared by comets, but Bennu’s bulk chemical and isotopic composition does not show clear evidence of a cometary component, such as depletion of the heavy Xe isotopes^50^.

Our analyses of aggregate samples indicate that Bennu’s parent body experienced substantial aqueous alteration but preserved enough pre-accretion components from diverse stellar, interstellar and Solar System sources to provide insight into its early formation environment. There are genetic similarities in the main rock-forming elements between Bennu, Ryugu and CI materials, but also distinctions. In particular, the analysed Bennu samples contain more anhydrous silicates and isotopically anomalous organic matter than samples of the hydrated Ryugu lithology^29,30,41^ and Orgueil^39^. This suggests that Bennu’s parent asteroid accreted a different mix of these materials than those of CIs and Ryugu. We propose that the parent bodies formed from a common reservoir beyond the snowline that was heterogeneous in space and/or time during the earliest evolution of the protoplanetary disk.

## Methods

### Samples

The samples studied (Supplementary Table 1) were derived from two sources: spillover on the avionics deck, outside the spacecraft’s Touch-and-Go Sample Acquisition Mechanism (TAGSAM)^51^ and from within the TAGSAM itself. Samples from the avionics deck were part of the ‘quick-look’ (QL) analysis phase of preliminary examination^2^ and have the ID structure OREX-5#####-0, where the hashtags represent a unique 6-digit numeric string. TAGSAM samples are denoted OREX-8#####-0. Subsamples have their own unique 6-digit string, whereas splits have the same 6-digit numeric string as their parent samples but with suffixes of -100, -101, -102 and so on, rather than -0. The QL samples were exposed to air during sample allocation, whereas TAGSAM samples were allocated under N_2_. All of the samples studied comprise aggregate material with particles sizes less than 0.5 cm in longest dimension^2^. All samples were transported from curation under N_2_ and were stored under N_2_ when not being studied.

Information on the samples studied, the elements and isotopes measured and in which laboratory can be found in Supplementary Table 1. The table also includes the DOIs of the data products underlying this work.

### Analytical techniques

#### Coordinated dissolution

An ~20.66 mg split of Bennu aggregate (OREX-803015-0) was dissolved at Washington University at St. Louis (WUSTL). Dissolution of the sample was done using concentrated HF and HNO_3_ in a 3:1 ratio for 48 h at 170 °C in a closed beaker, followed by fluxing the sample in concentrated HNO_3_ and HCl. While undergoing the HNO_3_ flux, 1 ml H_2_O_2_ was slowly added to the sample to remove organics. Once dissolution was complete, the sample were brought up in 5 ml 0.5 M HNO_3_. The solution was then split two ways: about half stayed at WUSTL and half was sent to Lawrence Livermore National Laboratory (LLNL). At LLNL, the aliquot was further split into two aliquots: one stayed at LLNL (OREX-803015-101) and the other was sent to ETH Zurich (OREX-803015-100).

#### Bulk elemental abundances

Bulk elemental abundances of OREX-803015-101 were determined at LLNL. Major and trace element concentrations were measured using a high-resolution ICP-MS (Thermo Element XR) at LLNL. A subaliquot of the bulk digest equating to approximately 0.5 mg of Bennu was dried down and redissolved in 5 ml internal standard solution. This consists of 2% HNO_3_ + 0.005 M HF, spiked with 1 ng g^−1^ of In, Re and Bi, which are used to correct for instrument drift and sample matrix effects. A series of solution standards and certified rock standards (US Geological Survey (USGS)) were prepared in parallel and diluted using the same internal standard solution. The Element ICP-MS was fitted with standard ‘H’ sample and skimmer cones, and solutions were aspirated using a 100 ml min^−1^ nebulizer (Glass Expansion). The Element was tuned for sensitivity and reduced oxides, with typical count rates between 1.2 × 10^6^ cps and 1.5 × 10^6^ cps for 1 ng g^−1^ of In, and oxide formation at ~5%. Most elements of interest were measured using low-resolution mode, but elements that are commonly subject to interferences, such as the transition metals, were measured at medium or high resolution (where low resolution is *R* = 300, medium resolution is *R* = 4,000 and high resolution is *R* = 10,000, with *R* = *m*/Δ(*m*), where *m* is mass). Sample count rates were background subtracted before quantification using a combination of reference solutions and rock standards. Accuracy was assessed using the USGS basalt standard BHVO-2, with most concentrations falling within 10% of reference values.

The two measurements (this study and ref. ^2^) were conducted by different laboratories using separate aliquots of the same solutions (this study at LLNL and data reported in ref. ^2^ at WUSTL). Minor differences in a few elements may stem from laboratory discrepancies, as the two labs use different calibration standards (geostandards versus synthetic standards) and different internal standards. Also, in the context of quadrupole ICP-MS analyses by different labs (and using different calibration standards), these two results are very close. Therefore, these small differences are likely not significant.

The Bennu data and reference data are plotted in Extended Data Fig. 1 and are tabulated in Supplementary Table 2 where the uncertainties provided are measurement errors (internal) at the 2σ level.

#### Bulk K, Cu and Zn isotopes

About 7 mg of sample OREX-803015-0 (total mass of 20.66 mg) was used for MVE isotope analyses at WUSTL. Dissolution of the sample was done using concentrated HF and HNO_3_ in a 3:1 ratio for 48 h at 170 °C in a closed beaker, followed by fluxing the samples in concentrated HNO_3_ and HCl. While undergoing the HNO_3_ flux, 1 ml H_2_O_2_ was slowly added to sample to remove organics. Potassium isotope separation was undertaken first using a triple-pass chromatography procedure with Bio-Rad AG50W-X8 100–200 mesh cation exchange resin (see ref. ^23^ for detailed description of the K separation procedure). Owing to limited sample mass, the separation of Cu and Zn was conducted on the matrix aliquots collected following K separation chemistry. The first pass of the Cu and Zn purification procedure was undertaken using AG1-X8 200–400 mesh anion exchange resin, whereby both elements were extracted one after the other (Cu was eluted using 22 ml of 6 M HCl, while Zn was eluted using 10 ml of 3 M HNO_3_). A second pass of the same procedure was undertaken to further purify Cu, while Zn was further purified using a procedure which still used AG1-X8 200–400 mesh anion exchange resin, but with 5 ml of 1.5 M HBr used to elute the matrix, and 3 ml of 0.5 M HNO_3_ to elute Zn (see ref. ^52^ for a detailed description of the Cu and Zn separation procedure).

The isotope analyses of K, Cu and Zn were all conducted using a Thermo Scientific Neptune Plus MC-ICP-MS. To lower the ArH^+^ peak and significantly increase the K signal intensity, all K isotope analyses were undertaken using a ‘dry plasma’ technique with the Elemental Scientific APEX Ω high-sensitivity desolvation system used as an introduction system (see ref. ^53^ for a detailed description of this technique). In addition, all K isotope analyses were undertaken using a high-mass-resolution slit. In contrast, Cu and Zn analyses were undertaken using a quartz glass dual cyclonic spray chamber introduction system and a low-mass-resolution slit.

To correct for instrument mass bias, the sample–standard bracketing technique was used for all analyses with NIST-SRM 3141a used as the K standard, NIST-SRM 976 used as the Cu standard and JMC-Lyon used as the Zn standard. The K isotopic composition is given as δ^41^K = ([(^41^K/^39^K)_sample_/(^41^K/^39^K)_standard_ – 1] × 1,000). The Cu isotopic composition is given as δ^65^Cu = ([(^65^Cu/^63^Cu)_sample_/(^65^Cu/^63^Cu)_standard_ – 1] × 1,000) and the Zn isotopic composition as δ^66^Zn = ([(^66^Zn/^64^Zn)_sample_/(^66^Zn/^64^Zn)_standard_ – 1] × 1,000). For both K and Zn, the analyses of samples and standards were conducted at a concentration of 200 ppb, while for Cu analyses were run at a concentration of 100 ppb. To monitor data quality, the geostandard BHVO-2 was analysed alongside all sample analyses.

Data are compiled in Supplementary Table 9. Non-Bennu data sources for Fig. 2 include δ^65^Cu data for CCs^21,22,54^ and non-carbonaceous chondrites (NCs)^54–56^, δ^41^K data for CCs^23,43,57–62^ and NCs^23,43,59–63^, and δ^66^Zn data for CCs^21,22,64–66^ and NCs^55,64,67,68^. Sources for non-Bennu elemental data include refs. ^22,41,57,69,70^.

#### Bulk Ti isotopes

Bulk Ti isotope analyses were conducted at two laboratories: Institute of Geochemistry and Petrology, ETH Zurich, Switzerland and LLNL, USA following coordinated dissolution (see above).

##### ETH Zurich

Bulk Ti isotope analyses were performed on a 5.2 mg aliquot of Bennu aggregate (OREX-803015-100) at ETH. Titanium was separated and purified through a three-step anion exchange chromatography procedure, following the method detailed by ref. ^71^. The total procedural blank for Ti was 3.7 ng, resulting in a maximum blank contribution of 0.18% for Ti. Yields of the purification procedure are 75–100%. High-precision Ti isotope data were measured using a Thermo Scientific Neptune Plus multi-collector inductively coupled plasma mass spectrometer at ETH Zurich, following ref. ^37^. The measurements were conducted at medium mass resolution, with a mass resolving power (*R*) of approximately 6,600 to 7,000 (*R* = *m*/*m*0.95 − *m*0.05)). Titanium isotopes were collected in two cup configurations. First, all five Ti isotopes and ^44^Ca were measured enabling correction of the Ca interference on ^46^Ti and ^48^Ti. The second configuration included ^49^Ti, ^50^Ti, ^51^V, ^52^Cr and ^53^Cr to correct for isobaric interferences from V and Cr on ^50^Ti. A sample measurement consisted of 40 cycles with 8.39 s integration time for the first configuration and 4.19 s for the second.

Each individual measurement consumed approximately 0.3 µg of Ti yielding a signal of around 40 V over a 10^11^-Ω resistor on ^48^Ti. To correct for instrumental mass bias, the isotope data were normalized to a ^49^Ti/^47^Ti ratio of 0.749766 (ref. ^72^), using the exponential law. The results are reported relative to an in-house Alfa Aesar Ti wire standard in the ε notation, applying the sample–standard bracketing method:

$${\upvarepsilon }^{i}{\mathrm{Ti}}=\left(\frac{{i/47\atop}{\mathrm{Ti}}_{{\mathrm{sample}}}}{{i/47\atop}{\mathrm{T{i}}}_{{{\mathrm{standard}}}}}-1\right)\times {10}^{4},$$

where *i* refers to the isotope masses ^46^Ti, ^48^Ti and ^50^Ti. The isotope data were collected on two different days and included four repetitions for Bennu. To verify the accuracy and reproducibility of these measurements, the terrestrial rock standard BHVO-2 and the Agua Zarcas (CM2) chondrite were analysed alongside the Bennu sample. The analytical uncertainties of 9 analyses of BHVO-2 are ±0.17 ε^46^Ti, ±0.09 ε^48^Ti and ±0.16 ε^50^Ti (2 s.d.).

##### LLNL

Bulk Ti isotope analyses were performed on an ~5 mg aliquot of Bennu aggregate (OREX-803015-101) at LLNL. Purification of Ti was performed using a three-stage separation procedure. First, Fe was separated using 7 M HCl–0.01% H_2_O_2_ and AG1-X8 (100–200 mesh) ion-exchange resin. Next, the cut containing Ti was converted to 12 M HNO_3_ and further purified following the methods outlined in refs. ^73,74^, using precleaned and preconditioned Eichrom DGA resin cartridges in combination with a vacuum box system. Finally, the Ti was further purified using 0.4 M HCl–1 M HF and AG1-x8 (100–200 mesh) ion-exchange resin. The USGS terrestrial rock standards BCR-2 and BHVO-2 were processed through the same chemical purification procedure to verify the accuracy of our methods. Yields of the purification procedure applied here are >90% and the total procedural blanks were 2 ng for Ti, which is negligible, given that >2 μg of Ti were processed from our aliquot of Bennu.

Titanium isotope measurements were completed using the Thermo Scientific Neoma with an Aridus II and Jet sampler and X skimmer cones. All five Ti isotopes as well as ^44^Ca, ^45^Sc, ^51^V, ^52^Cr and ^53^Cr were collected in one line using Faraday cups (FCs) connected to 10^11^-Ω resistors. All samples and standards were measured on the flat low-mass peak shoulders in medium-resolution mode to avoid molecular interferences. Samples were bracketed with the Origins Lab Ti standard and were measured at concentrations of 200 ng g^−1^ Ti, resulting in intensities of ~40 V on ^48^Ti. Data were normalized to ^49^Ti/^47^Ti = 0.749766 and collected with 50 cycles with 4 s integration time each. The analytical uncertainties of these methods as determined from 16 analyses of BCR-2 and BHVO-2 are ±0.29 ε^46^Ti, ±0.16 ε^48^Ti and ±0.26 ε^50^Ti (2 s.d.).

It should be noted that masses 44 (Ca), 45 (Sc), 51 (V), 52 and 53 (Cr) were monitored during the Ti isotope measurements to monitor potential isobaric interferences from other elements. However, due to the effective chemical isolation, these signals were always close to or indistinguishable from background. The corrections based on these signals are well within the limits that have been previously shown to be accurate.

Data are compiled in Supplementary Table 10. The sources of non-Bennu data in Fig. 3 include Ti data^37,71,74–89^ and O data^25,26,90–97^.

#### Bulk anion abundances by ion chromatography

A 25.6 mg Bennu aggregate (OREX-803001-0) was sealed in a glass ampoule with 1 ml Milli-Q ultrapure water and heated at 100 °C for 24 h. The sample was centrifuged and the supernatant was separated from the solid residue. Forty per cent of the extract was dried, acid-hydrolysed under 6 M HCl vapour at 150 °C for 3 h and desalted by passing the solution through an ion-exchange chromatography column (acid-hydrolysed wash, OREX-803001-111). Murchison acid-hydrolysed wash and procedural blank were prepared the same way. The solutions were transferred to the Astromaterials Research and Exploration Science Division (ARES)/Johnson Space Center (JSC) Analytical Geochemistry Lab for anion analysis by ion chromatography. Anions were analysed by a multi-gradient method at flow rate 2 ml min^−1^ using a Dionex Integrion instrument equipped with a Dionex IonPac AS11 4 × 250 mm column, the Dionex EGC 500 KOH eluent generator cartridge and a Dionex DRS 600 dynamically regenerated suppressor with a 20 µl injection volume. Samples were analysed for acetate, formate, Cl^−^, SO_4_^2−^, PO_4_^3−^, F^−^, Br^−^ and NO_3_^−^. Results were corrected against a procedural blank. The results are reported in Supplementary Table 3. While the abundance of chloride noted in Supplementary Table 3 is high, it is important to note that it originated from the HCl that was used for hydrolysis and not from the sample.

All published sulfate data^98–100^ shown in Extended Data Fig. 2 were measured with ion chromatography. The analysed samples were water extracts from meteorites and Ryugu. All extractions, except the one in ref. ^98^, were done under conditions similar to the methods used for Bennu (ref. ^99^, 20 h at 100 °C; ref. ^98^, 25 h at room temperature; ref. ^100^, 20 h at 105 °C; Bennu samples and our previous unpublished data, 24 h at 100 °C). The actual ion chromatography procedures to measure dissolved anions differed because different instruments, columns, eluent solutions and so on were used.

#### Bulk O isotopes

Oxygen isotopic analyses were undertaken at the Open University (OU, Milton Keynes, UK) using an infrared laser-assisted fluorination system. An ~150 mg sample of Bennu aggregates (OREX-800032-0) was transported from the JSC Curation Facility to the Natural History Museum (NHM) in London in glass dimple slides sealed in a N_2_ atmosphere within an Eagle sample container. A randomly selected ~15 mg subsample (OREX-803099-0) was prepared in the N_2_ glovebox at the NHM and transferred to the OU in dimple slides in a N_2_ atmosphere within the Eagle container. The sample was then stored and processed in the N_2_ glovebox at the OU, ensuring that the sample was protected from atmospheric exposure at all stages from departing JSC curation to analysis.

Four subsamples of OREX-803099-0 were prepared for oxygen-isotope analyses (a further two were prepared for the stepped heating C and N measurements also reported here). OREX-803110-0 (2.3 mg) and OREX-803140-0 (3.3 mg) were randomly selected splits considered representative of the overall sample. An aluminium foil strip was used as a brush to preferentially select coarser or finer particles within the aggregate to produce samples OREX-803136-0 (2.2 mg of coarser particles) and OREX-803137-0 (2.4 mg of finer particles). The range in particle size was not large, with the typical particle size diameter in the two samples estimated at ~400 µm and ≤200 µm, respectively. Sample masses are provided as a guide, but the challenges of weighing small samples in our glovebox creates considerable uncertainty (estimated at ~20%).

The laser fluorination measurements were made at the OU and are based on the established methods developed for the analyses of primitive chondritic materials with high volatile and/or organic contents (typically CI- and CM-like CCs) and used for the study of Ryugu samples^26^. The method employs a ‘single shot’ approach, whereby only one sample is loaded into the sample tray in a N_2_ glovebox, with the sample chamber baked and pre-fluorinated before transfer to the glovebox.

In brief, the single-shot method involved admitting an aliquot of BrF_5_ into the sample chamber at room temperature for 5 min. For the analysis of meteorites and other samples exposed to the terrestrial atmosphere, this step is used is to remove any residual moisture or O_2_ adsorbed on to the sample chamber walls or sample, although, as per usual, some reaction of the sample also occurs. However, the samples analysed in this study have been protected from the terrestrial environment at all stages, except for a few tens of minutes during Sample Return Capsule (SRC) entry and decent and recovery of the capsule (but all moisture should have been removed by the SRC filter system before any brief exposure). The oxygen gas liberated in this pre-fluorination step had isotopic signatures very similar to the laser-assisted fluorination step that followed, and therefore the isotopic measurements were combined to provide a bulk measurement. Following the pre-fluorination, the sample itself was reacted by heating in the presence of BrF_5_ with a Photon Machines 50-W infrared CO_2_ laser (10.6 µm). Liberated O_2_ from each step in the analysis was purified, including removal of NF_3_ on 13X molecular sieve at −130 °C before being admitted to the inlet system of the mass spectrometer for analysis. The isotopic composition of the purified oxygen gas was analysed using a Thermo Fisher MAT 253 dual-inlet mass spectrometer. Sample gas/reference gas comparisons were performed for 30 min, with rebalancing every 10 min. A mass scan over *m*/*z* = 52 was conducted on each sample to check that no NF_2_ fragment ions of NF_3_ were present. The errors quoted for individual measurements are 2 s.e. on the mean of the sample–standard comparisons. The results were corrected for a small blank, typically amounting to <2% of the total O_2_ analysed.

The total amount of oxygen liberated from the two fluorination steps is estimated at approximately 15 wt%—about 50% of the expected yield, although there is some uncertainty about the accuracy of these values because of the challenges of weighing small samples in a glovebox, where the balance conditions are not optimized. However, CI meteorites weighed under optimal conditions also provide low yields, typically 17 wt% O (ref. ^26^). The difference with Bennu samples is believed to be related to the additional oxygen present in the meteorites as a result of formation of ferrihydrite and sulfates through interaction with Earth’s atmosphere, as these phases have not been observed in either the Ryugu or Bennu samples, plus the abundant interlayer water present in CIs^9^. While the low yield has the potential to induce unwanted isotopic effects, the high temperatures associated with the laser-assisted fluorination should minimize any isotopic fractionation effects. Comparing laser-assisted fluorination of CI meteorites^26^ with those performed by fluorination bomb reaction techniques^25^ indicate no discernible difference in the reported isotopic composition of such samples.

Oxygen isotopic analyses are reported in standard δ notation, where δ^18^O has been calculated as:

$$\updelta^{18}{\rm{O}}(\permil)=\left[\left({{\scriptstyle{18}}\atop}{\rm{O}}/{{\scriptstyle{16}}\atop}{\rm{O}}\right)_{{\rm{sample}}}/{\left({{\scriptstyle{18}}\atop}{\rm{O}}/{{\scriptstyle{16}}\atop}{\rm{O}}\right)}_{{\rm{VSMOW}}}-1\right]\times 1,000$$

and similarly for δ^17^O using the ^17^O/^16^O ratio. VSMOW is the international standard, Vienna Standard Mean Ocean Water. Δ^17^O represents the deviation from the terrestrial fractionation line and has been calculated as:

$$\Delta {}^{17}{\rm{O}}=\updelta {}^{17}{\rm{O}}-0.52\times \updelta {}^{18}{\rm{O}}$$

Analytical precision for sample sizes comparable to those used in this study, as defined by replicate analyses of our internal obsidian standard, is: ±0.05‰ for δ^17^O; ±0.10‰ for δ^18^O; ±0.02‰ for Δ^17^O (2 s.d.)^101^.

The bulk values for the TAGSAM material are similar to those obtained for aggregate samples collected from the avionics deck as part of the QL study (average δ^18^O = 20.6 ± 2.7‰, and Δ^17^O = 0.72 ± 0.16‰ (2 s.d.))^2^, despite these initial analyses being performed on samples exposed to air for several weeks before analysis and not including the pre-fluorination step. The variation in δ^18^O in the Ryugu samples appears to result from mineralogical control, exacerbated by the very small sample size used for some of these samples^26^. Very little variation is observed in the results from the samples reported here, although one of the replicates of the sample (OREX-803110-0) had a measurably different Δ^17^O value that appears to indicate the presence of a rare grain with distinct oxygen isotopic composition. CI chondrites contain abundant interlayer water with a terrestrial O-isotope signature^26^ whereas Ryugu samples contain very little interlayer water^9^ (the amount of interlayer water in Bennu samples has not been reported yet). These modifications likely lead to a significant shift in the bulk O-isotope composition to lower Δ^17^O and δ^18^O (ref. ^26^).

Bennu data are compiled in Supplementary Table 4. Non-Bennu data shown in Extended Data Fig. 3 are from refs. ^9,25,26,102,103^. The CC anhydrous mineral line (Fig. 5 and Extended Data Fig. 3) and primitive chondrite minerals line (Fig. 5) are constructed from refs. ^104^^,^^105^, respectively.

#### Bulk C and N abundances and isotopes

The samples analysed at the OU were separated under N_2_ at the JSC, sealed and hand-carried to the NHM in London. Still under N_2_ in a glovebox, the OU allocation was weighed, then again sealed and hand-carried to Milton Keynes, where it was again placed in a glovebox under N_2_. The first sample (OREX-803058-0, 1.427 mg) was weighed into a cleaned Pt envelope (25 µm thick, 99.9% purity Johnson Mattey Pt foil; cleaned by combustion at 1,200 °C) on a microbalance in the glovebox, then transferred into a portable vacuum manifold that was then attached to the extraction system of the OU’s Finesse mass spectrometer system^106–108^. This sample was not exposed to air before analysis. The second sample (OREX-803059-0, 1.170 mg) was transferred from the OU glovebox to a class 100 clean room, where it was weighed into a Pt envelope before admission to the Finesse system. This sample was exposed to air in the clean room; there were, however, no significant differences in the results at the lowest temperatures of the analysis that could be ascribed to adsorbed terrestrial atmosphere.

The main feature of the fully automated Finesse system is its ability to analyse simultaneously the abundances and isotopic compositions of several light elements (He, C, N, Ne, Ar and Xe) extracted from a single sample. Finesse consists of 2 triple-collector 12 cm magnetic sector noble gas-type static mass spectrometers plus a quadrupole mass spectrometer, all coupled to a common extraction system. One of the magnetic sector mass spectrometers is used for the analysis of carbon as CO_2_; the other for molecular N_2_ and Ar. The quadrupole spectrometer is used for He, Ne and Xe. Only C and N data are reported here.

The sample in its Pt envelope was introduced to a double-walled combustion tube (inner wall of quartz glass and outer wall of corundum separated by a vacuum gap) within a SiC furnace. It was evacuated to a pressure of ~10^−8^ mbar then heated to either 50 °C or 100 °C under vacuum to remove adsorbed terrestrial species. The experiment then proceeded by heating the sample in increments to 1,450 °C under pure oxygen (generated by heating CuO to 850 °C) in the presence of a Pt catalyst (also maintained at 850 °C).

At the end of the combustion step, excess oxygen was resorbed by copper oxide at 450 °C. Oxygen pressure during oxidation was 5–10 mbar and the combustion time was 0.5 h. The products of combustion (CO_2_, N_2_, SO_2_, H_2_O and noble gases) were separated using a series of cryogenic traps. CO_2_, SO_2_, H_2_O and Xe were trapped in a glass finger. N_2_ and Ar were adsorbed onto a finger containing a 5 Å zeolite molecular sieve, while He and Ne remained in the gas phase. Controlled heating of the cold fingers enabled individual species to be isolated for additional purification and quantification. The noble gases were held over an Al–Ti getter for 10 min; N_2_ was held over a second Cu/CuO finger and Pt catalyst for 20 min, to ensure reduction of any nitrogen oxides to N_2_. Water and SO_2_ could not be measured quantitatively on the system so were pumped away. The amount of CO_2_ was measured using a capacitance monometer (BaratronTM) with a precision better than 1%; amounts of the other gases were determined from calibration of the ion beam current, knowing the volumes of all the different sections of the extraction manifold into which the gases were expanded.

The noble gas-type mass spectrometers for N_2_ and CO_2_ are each equipped with three collectors set for masses of 28, 29, and 30 and 44, 45, and 46, respectively. The measurement itself took approximately 1 min, during which ~300 data points were collected for each isotope, providing a precision of 0.3–0.5‰. A volume of laboratory standard gas equivalent to that of the sample was measured between each set of data points to enable calculation of isotopic composition. The standards were calibrated using either National Bureau of Standards reference materials (calcite for CO_2_) or atmospheric nitrogen (for N_2_) taken from a fixed-volume gas pipette system. The sampling system for noble gas standards (air) is similar and also calibrated in an appropriate manner.

System blank was determined by the analysis of an empty Pt foil envelope; the amount of gas in the blank depends on temperature, hence the blank experiments covered the same temperature range as the samples. At the highest temperatures of the analyses, where the smallest quantities of gas were released from the sample, the blank contribution (~0.5 ng for N_2_; ~20 ng for CO_2_) was still less than 10% of the sample, so blank contributions were not significant.

Data are compiled in Supplementary Table 5. Non-Bennu data presented in Extended Data Fig. 4 are from the OU, apart from Ryugu data^18,109,110^.

#### Bulk noble gases

Noble gas analyses were conducted at three laboratories. He, Ne, Ar and Xe analyses were performed at Centre de Recherches Pétrographiques et Géochimiques (CRPG), Nancy, France, and Institute of Geochemisty and Petrology, ETH Zurich, Switzerland; additional Xe analyses were conducted at the Department of Earth and Environmental Sciences, The University of Manchester, UK. Data are compiled in Supplementary Tables 6–8 for Ar, Ne, and Xe, respectively.

The He, Ne, Ar, Kr and Xe isotope composition of 8 particles from asteroid Bennu, weighing 0.095–1.42 mg, were analysed using an all-noble-gas analytical system installed at CRPG. Particles were handpicked from aggregate sample OREX-800032-100 in a clean room (ISO6) at CRPG. The particles were briefly exposed to air for precise weighing before being placed into different pits of a laser chamber, which was baked at 100 °C and pumped down to 10^−9^ mbar overnight to remove any adsorbed atmospheric gases. Each particle was then sequentially heated using a CO_2_ laser working at 10.6 µm. After each incremental increase in laser power, extracted gases were purified, cryogenically separated and analysed on the Helix MC^+^ (Thermo Scientific) following previously established protocols^17,111^. Here we present the bulk analysis of Ne and Xe in sample OREX-800032-105, which was the largest grain analysed at CRPG.

The 3 aggregate samples OREX-800032-102, OREX-800032-103 and OREX-800032-104 of 0.9396 ± 0.0003, 0.8901 ± 0.0006 and 0.0678 ± 0.0006 mg mass, respectively, were received at ETH Zurich from the NHM in London. They were weighed and loaded into the ultra-high vacuum (UHV) system all within N_2_ atmosphere to minimize atmospheric noble gas contamination. Gas extraction was achieved by heating the samples individually for 2 min by infrared laser (continuous-wave Nd:YAG Spectron SL902TQ laser emitting at 1,064 nm with a maximal power of 65 W) at 82–87% in 2 extraction steps until the samples were fused to glass beads. The respective second step confirmed complete gas extraction in each first main step. Sample gas cleaning, separation into He–Ne, Ar and Kr–Xe fractions, and measurements in an in-house built sector field mass spectrometer ‘Albatros’, equipped with a highly linear Baur-Signer ion source, a multiplier operated in ion-counting mode and an FC are detailed in refs. ^13,112^. Blanks were measured by heating the Al sample holder without sample under the same conditions as the samples. Blank corrections for the main steps of the two 0.9 mg samples amounted each to <1% for all isotopes except for ^40^Ar (15–22%). Blank corrections for the 68 µg sample were <1.5% for He, ^36,38^Ar and Xe isotopes, <7% for Kr, ~11% for Ne and ~19% for ^40^Ar. Here we present the Ar, Ne and Xe data. The source data for Fig. 1 includes Ryugu data^18^, and data from CI^19,75,112–115^, CM^20^, CR^116^ and CO^117^ chondrites.

The Xe isotopic composition of sample OREX-803060-0 (~60 µg) was analysed using the RELAX^118,119^ mass spectrometer at the University of Manchester. The sample was too small to weigh using the balances available. The mass was estimated using images taken with an optical microscope before analysis. The particle was assumed to be an ellipsoid, the volume estimated from measurements of the 3 perpendicular axes, and the mass then calculated using the initial density estimates^120^ of between 1.5 g cm^−3^ and 1.8 g cm^−3^. The normal procedure for loading samples into a noble gas mass spectrometer involves evacuating the extraction line and sample port and then baking them to temperatures ~180 °C. We did not bake the sample port, to allow us to investigate any low-temperature gases that might be lost from the sample during baking^121^. After loading samples, the sample port and extraction line were both evacuated, the port was then isolated from the line, and just the extraction line was baked. The sample port was then pumped for ~2 weeks at room temperature to preserve low-temperature components. Analyses then proceeded following previously published methods^18,118^.

#### Isotope mapping for presolar grains and organic matter

In situ isotope mapping was conducted at two laboratories: ARES at NASA JSC and the Lunar and Planetary Laboratory, University of Arizona (UA), Tucson, USA. Organic matter was characterized at NASA JSC.

##### NASA JSC

Sample OREX-501018-100 consisted of aggregate QL material pressed onto a gold (Au) foil mount using a clean sapphire window. The Au foil had been annealed and HF-cleaned and was mounted onto an Al stub. The CAMECA NanoSIMS 50L was used to search for presolar grains and isotopically anomalous organic matter in this sample by raster ion imaging. The isotopic standards used to correct for instrumental mass fractionation were USG24 graphite, KG17 kerogen and San Carlos olivine. These standards were prepared in the same manner as the OREX-501018-100 sample. The δ^13^C value of USG24 is −16.05‰. KG17 has a δ^13^C value of −24.1‰, a δ^15^N value of 5.2‰ and a δD value of −108‰. San Carlos olivine has δ^17^O and δ^18^O values of 2.73‰ and 5.25‰. The isotopic compositions of these standards, and those reported for the presolar grains and organic matter in Bennu, are relative to standard mean ocean water (SMOW) for O and H, Pee Dee Belemnite (PDB) for C, and atmospheric N_2_ for N.

The CAMECA NanoSIMS 50L at NASA JSC was used to search for presolar grains and isotopically anomalous organic matter in OREX-501018-100 by raster ion imaging. An ~1.8-pA, ~150-nm-diameter primary beam was rastered over regions of interest. The C and N (measured as ^12^CN) isotopes, ^28^Si, ^30^Si, and ^32^S were measured simultaneously as negative ions in electron multipliers (EMs). In a subsequent session, the C and O isotopes, ^28^Si, and ^24^Mg^16^O were measured using an ~0.9-pA, ~100-nm Cs^+^ primary beam. H isotopes, ^13^C and ^18^O were then measured using an ~14-pA primary beam. The mass resolving power of ~10,000 (CAMECA NanoSIMS definition^122^) allowed for resolution of isobaric interferences, particularly on masses ^13^C, ^17^O and ^12^C^15^N.

Each 20 × 20 µm^2^ region of analysis was first pre-sputtered, over areas of 22 × 22 µm^2^, using a 16-keV Cs^+^ primary ion beam of high current (~180 pA) to clean the sample surface, implant Cs^+^, and ensure that secondary ion count rates reached a steady state. An electron flood gun (~300 nA) was used to mitigate sample charging. An ~1.8-pA, ~150-nm-diameter primary beam was rastered over the regions, which consisted of 256 × 256 pixels. The C and N (measured as ^12^CN) isotopes, ^28^Si, ^30^Si, and ^32^S were measured simultaneously as negative ions in EMs. Each ion image consisted of 256 × 256 pixels, which were analysed at 3,000 μs per pixel for 40 frames. In a subsequent session in regions that were not previously measured, the C and O isotopes, ^28^Si, and ^24^Mg^16^O were measured using an ~0.9-pA, ~100-nm Cs^+^ primary beam. Each ion image consisted of 256 × 256 pixels, which were analysed at 4,200 μs per pixel for 40 frames. H isotopes, ^13^C and ^18^O were then measured using an ~14-pA primary beam. Multiple frames were acquired for each analysis region. Each ion image consisted of 256 × 256 pixels, analysed at 1,800 μs per pixel for 32 frames.

The C, N and O isotopic ratios were corrected for instrumental mass fractionation using USG24 graphite, KG17 kerogen and San Carlos olivine, respectively. Kerogen was also used to correct the H-isotope ratios. The ^30^Si/^28^Si ratios were normalized to the Si-rich material that was not isotopically anomalous. Data processing was conducted using the L’Image software (developed by L. Nittler). Grains were considered presolar if their isotopic composition differed from the reference ratios by >5*σ* and if the isotopic anomaly was present in multiple consecutive frames (Supplementary Table 11). Preliminary phase identifications were made based on the NanoSIMS ^28^Si/^12^C, ^28^Si/^16^O and ^24^Mg^16^O/^16^O ratios. Grains with Si/C ratios >0.2 were considered to be SiC and grains with Si/C ratios <0.2 were classified as graphite. Presolar grains with Si/O ratios similar to the surrounding matrix (~0.01), which is dominated by silicates, were considered to be silicates and grains with low Si/O ratios (<0.001) were oxides. Two O-rich presolar grains were also analysed by SEM-EDS to further constrain the phase and to confirm the phase identifications made based on the NanoSIMS data. Organic grains were defined by manual and automated means and were considered isotopically anomalous, relative to the bulk composition, if they deviated by >3*σ* from the average (bulk) isotopic compositions. Abundances of isotopically anomalous organic matter are given in area% (area of anomalous organics divided by total area analysed; Supplementary Table 13).

Presolar grain abundances are reported as parts per million (ppm) and include all grains identified at NASA JSC and at UA (Supplementary Table 12). The abundance of each presolar phase (SiC, graphite and O rich) was determined by dividing the summed area of the presolar phase by the total area of material analysed. These areas were assessed from the NanoSIMS ion images. The total area analysed was determined by placing thresholds on the ^16^O, ^28^Si and ^12^C images (pixels with low counts were excluded). The total areas mapped for C and O isotopes was 25,794 μm^2^, and for C and N isotopes was 8,323 μm^2^. Abundances of isotopically anomalous organic matter are given in area% (area of anomalous organics divided by total area analysed). The total area measured for C and N isotopes was 8,323 μm^2^. For H isotopes, the threshold was placed on the H maps and the total area measured was 7,053 μm^2^.

##### University of Arizona

Samples OREX-501049-100 and OREX-501080-0 were prepared at the UA. OREX-501049-100 was prepared by pressing aggregate particles into Au foil on top of an Al stub. This sample was not polished. OREX-501080-0 was prepared as a polished section by embedding aggregate particles in Struers epoxy. This sample was ground dry using SiC paper and polished dry using diamond paste. The sample was cleaned only using compressed air and white paper shop towel.

A terrestrial kerogen standard deposited onto Au foil was used for tuning and to correct instrumental mass fractionation for C and N isotopes, and surrounding matrix was used to normalize O isotopes assuming Solar System values (SMOW). The terrestrial kerogen is from chert of the Warrawoona group (002-1-RK-M) with a δ^13^C value of −34.3‰ and a δ^15^N value of ~2‰, relative to PDB and atmospheric, respectively. It is a well-characterized standard used for over a decade at WUSTL as tuning and reference material for NanoSIMS and Auger nanoprobe work (for example, ref. ^123^).

Bennu samples were imaged using the Keyence VHX7000 digital optical microscope. Reflected light whole-sample maps were produced to aid navigation in subsequent instruments. Both samples were coated with carbon before SEM and NanoSIMS analysis. Both samples were examined in the Hitachi TM4000plus scanning electron microscope using a 15-keV electron beam. Backscattered electron mosaic images of the samples were collected to identify suitable fine-grained matrix areas for subsequent isotopic analysis.

Isotopically anomalous grains were located in OREX-501049-100 and OREX-501080-0 using the CAMECA NanoSIMS-High-Resolution in the Kuiper-Arizona Laboratory for Astromaterials Analysis (K-ALFAA). Both samples were coated with carbon before analysis. We carried out raster ion imaging using a focused Cs^+^ primary beam of ~1–1.2 pA and ~100 nm in diameter. An electron flood gun was not used. Secondary ions of ^12,13^C^−^, ^16,17,18^O^−^ and ^12^C^14,15^N^−^, and secondary electrons, were simultaneously acquired in multi-collection mode. The mass resolving power was between 9,000 and 12,000 for all detectors (CAMECA definition^122^). To remove the carbon coat and to implant primary ions, we first rastered a high beam current (~150p A) over 11 × 11 µm^2^ areas on the NanoSIMS-High-Resolution. Each measurement then consisted of 10–20 scans of 10 × 10 µm^2^ (256 × 256 pixels) areas within the pre-sputtered region, with dwell times of 10,000–15,000 µs per pixel.

C, O and N isotope data were processed using the WinImage from CAMECA and L’Image software. A grain was considered presolar if its isotopic compositions deviated from the average surrounding material by more than 4*σ*, and if the anomaly was present in at least 3 consecutive frames. While the thresholds for presolar grain identification differ between the UA and JSC labs, previous studies have independently reported similar abundances for the same meteorites using these different thresholds. For example, in ALHA 77307, ref. ^124^ reported a presolar silicate abundance of 161 ± 16 ppm and ref. ^125^ of 171 ± 21 ppm.

Presolar grain abundances are reported as parts per million (ppm) and include all grains identified at NASA JSC and at UA. The abundance of each presolar phase (SiC, graphite and O rich) was determined by dividing the summed area of the presolar phase by the total area of material analysed. These areas were assessed from the NanoSIMS ion images. The total area analysed was determined by placing thresholds on the ^16^O, ^28^Si and ^12^C images (pixels with low counts were excluded). For H, the threshold was placed on the H maps. The total area mapped for O isotopes was 42,900 μm^2^ and for C and N isotopes was 43,600 μm^2^. As Si isotopes were not measured at UA, the UA C-rich presolar grains are assumed to be SiC.

In Fig. 4, Ryugu data are from refs. ^30,39^ and CI and CM chondrites^38,126–128^. Data on presolar grain isotopic compositions, presolar grain abundances and the compositions of organics are compiled in Supplementary Tables 11–13, respectively.

#### In situ chemical composition and O isotopes of anhydrous minerals

In situ O-isotope analyses were made at three different laboratories: CRPG, Nancy, France; Isotope Imaging Laboratory (IIL), Hokkaido University, Sapporo, Japan; and Planetary and Space Sciences at the OU, UK. All data are compiled in Supplementary Table 14. Non-Bennu data in Fig. 5 are from ref. ^33^.

##### Centre de Recherches Pétrographiques et Géochimiques

Samples OREX-800045-103 and OREX-800045-107 were prepared by Guy Liborel at Université Côte d’Azur. Aggregate particles (<1 mm) were mounted in epoxy, polished and were subsequently carbon coated.

SEM observations were performed on the samples using a JEOL JSM-6510 with 3-nA primary beam at 15 kV. We also performed multi-element EDS mapping (Mg, Si, Fe, Ni, S, Na, Ca and Al) of the different grains. Quantitative chemical analyses were performed using a JEOL JXA-8230 electron microprobe analyser (EPMA) equipped with five wavelength-dispersive spectrometers and one silicon drift detector energy dispersive spectrometer. Quantitative analyses were performed with an accelerating voltage of 20 kV, a probe current of 10 nA and beam diameter of 1 µm. For carbonates, we rastered the beam over 5 × 5 µm^2^. We used two different settings to determine the chemical compositions of minerals: (1) Al, Ti, Ca, Cr, Mn, Ni, Mg, Fe and Si (session 1) and (2) Na, K, Al, Ti, Ca, Cr, Mn, Ni, Mg, Fe and Si (session 2). We used different standards for tuning the EPMA: springwater olivine (Mg, Si), fayalite (Fe), wollastonite (Ca), albite (Na, Al), orthoclase (K), rutile (Ti), Ni metal (Ni), chromite (Cr) and rhodochrosite (Mn). The total peak + background counting time was 200 ms for Al, Ti, Ca, Mn and Cr, and 20 ms for Mg, Fe and Si. Detection limits were 0.025 wt% (Mg), 0.025 wt% (Fe), 0.05 wt% (Si, K, Na), 0.005 wt% (Ca), 0.02 wt% (Al), 0.005 wt% (Ti), 0.015 wt% (Cr) and 0.008 wt% (Mn).

Oxygen isotopic compositions of olivine and pyroxene were measured in OREX-800045-103 and OREX-800045-107 during two analysis sessions by SIMS using a CAMECA IMS 1270 E7 at CRPG-CNRS^129^. ^16^O^−^, ^17^O^−^ and ^18^O^−^ ions produced by a Cs^+^ primary ion beam (~1.5 µm, 30 pA) were measured in multi-collection mode using off-axis FCs for ^16^O^−^, the axial EM for ^17^O^−^ and an off-axis EM for ^18^O^−‍^. To remove ^16^OH^−^ interference on the ^17^O^−^, peak and achieve maximum flatness atop the ^16^O− and ^18^O^−^ peaks, the entrance and exit slits of the central EM were adjusted to achieve a mass resolving power (MRP = *M*/Δ*M*) of ~7,000 for ^17^O^−^ (CAMECA definition^122^). The multi-collection FC was set on exit slit 1 (MRP = 2,500). The total measurement duration was 20 min, comprising 10 min of pre-sputtering and 10 min of measurement.

Five terrestrial standard materials (San Carlos olivine, Dolomine dolomite, JV1 clinopyroxene, Saint-Paul enstatite and Rockport fayalite) were used to define the instrumental mass fractionation line for the three oxygen isotopes and correct for instrumental mass fractionation due to matrix effects in olivine.

To monitor any instrumental drift and to achieve good precision, the San Carlos olivine was analysed before and after every series of 10–15 sample analyses. To monitor any instrumental drift and to achieve good precision, the San Carlos olivine or the JV1 clinopyroxene were analysed before and after every series of 10–15 sample analyses. We measured the oxygen isotopic compositions of seven isolated olivine in three different particles of OREX-800045-103. We also measured the oxygen isotopic compositions of ten isolated olivine and one pyroxene grains in two different particles of OREX-800045-107. We additionally performed five analyses on matrix for reference.

To precisely localize the small olivine grains (~10 µm), barely visible on the CAMECA IMS 1280-HR SIMS charge-coupled device camera, we first made a few sputtered craters near the supposed locations of the targets using the 30-pA Cs beam and imaged the area with a scanning electron microscope following the method described in ref. ^129^. Using ^16^O^−^ ion images, we then localized the craters and calculated the position of the olivine targets using the SEM images. Oxygen isotopic compositions are expressed in δ notation as δ^17,18^O = ([^17,18^O/^16^O]sample/[^17,18^O/^16^O]VSMOW − 1) × 1,000‰. Samples related by mass fractionation to the VSMOW composition plot along a line with a slope of 0.52, defining the terrestrial fractionation line, whereas mass-independent variations are described by Δ^17^O = δ^17^O − 0.52 × δ^18^O, representing vertical deviations from the terrestrial fractionation line in a triple-oxygen-isotope diagram. Typical 2*σ* uncertainties, accounting for internal errors on each measurement and the external reproducibility of the standard, were estimated to be (1) ~0.5‰ for δ^18^O, ~0.6‰ for δ^17^O and ~0.6‰ for Δ^17^O (session 1) and (2) ~1.1‰ for δ^18^O, ~0.8‰ for δ^17^O and ~0.9‰ for Δ^17^O (session 2). The error on Δ^17^O was calculated by quadratically summing the errors on δ^17^O and δ^18^O. All SIMS analytical spots were checked thoroughly by SEM, and any spots near fractures or not completely within olivine/pyroxene grains were excluded from the data set.

##### Hokkaido University, Japan

A polished section of OREX-803114-0 was used for mineralogical and petrological observations and in situ O-isotope measurements by SIMS. The sample preparation procedure was established by ref. ^33^. The ten Bennu grains were embedded in a one-inch epoxy disk using the Buehler EpoxiCure 2 Resin. After embedding, its sample surface side was also impregnated with the resin in vacuum, to avoid collapsing the fragile samples during polishing. The sample disk was polished with an automatic polishing machine (Musashino Denshi MA-200e) at Hokkaido University. Diamond slurry with polycrystalline diamond particles of ~3 µm dissolved in ethylene glycol sprayed on a copper polishing plate was used to obtain flat surface of the sample disk. During the flattening, the sample surface was impregnated with the resin in vacuum a few times. Subsequently, ~1 µm diamond slurry sprayed on a tin–antimony alloy polishing plate and on polishing cloth were used to finalize the polishing. Only >99.5% ethanol was used for cleaning during and after the polishing. The polished sections were coated with a thin (~20 nm) carbon film for backscattered electron and X-ray imaging, and elemental analysis before in situ O-isotope measurements.

Backscattered electron images were obtained using a field-emission scanning electron microscope (JEOL JSM-7000F) at Hokkaido University. X-ray elemental analyses were conducted with a 15-keV electron beam using an EDS (Oxford X-Max 150) installed on the field-emission scanning electron microscope. Beam currents of ~2 nA and ~1 nA were employed for the X-ray mapping and quantitative analysis, respectively. Quantitative calculations were conducted using Oxford AZtec software. X-ray elemental maps covering the entire polished section of OREX-803114-0 were obtained with pixel size of 0.24 µm to systematically find olivine and pyroxene grains that can be measured for O isotopic compositions with SIMS. After electron microscopy was completed, the polished sections were recoated with an additional thin (~70 nm) Au film for SIMS measurements. The O isotopic compositions of 58 grains of olivine and 7 pyroxenes in OREX-803114-0 were measured in situ with the CAMECA ims-1280-HR SIMS instrument at Hokkaido University. The analytical and instrumental settings were established by ref. ^130^ and were similar to those described in ref. ^33^.

In detail, a ^133^Cs^+^ primary beam accelerated to 20 keV was employed. Negative secondary ions (^16^O^−^, ^17^O^−^ and ^18^O^−^) were measured simultaneously in the multi-collection mode. The mass resolution of *M*/Δ*M* for ^17^O^−^ was set at >6,000 to resolve ^17^O^−^ from ^16^OH^−^, while that for ^16^O^−^ and ^18^O^−^ was ~2,000 (CAMECA definition^122^). The automatic centring program was applied before data collection. A normal-incidence electron flood gun was used for electrostatic charge compensation of the analysing areas during the measurements. Analysed areas were precisely determined according to scanning ion image of ^16^O^−^ collected by a multi-collector EM (designated as L2), which was not used for the data collection, using a procedure established in ref. ^131^. Before measurements, we made a few sputtered craters near measurement targets using an ~30-pA primary beam by the SIMS and then acquired electron images with the field-emission scanning electron microscope to obtain distances from the sputtered craters to the measurement targets. The craters were visible in ^16^O^−^ scanning images and were used to locate the target minerals.

The reported uncertainties in the O isotopic compositions were the larger of the external reproducibility of standard measurements (2 s.d.) or internal precision (2 s.e. of cycle data) of samples. Measurement spots were observed by the field-emission scanning electron microscope after SIMS measurements. The data from spots with inclusions and overlapping matrix minerals were rejected.

We used two conditions with different primary beam currents depending on mineral sizes. An ~1.5-nA primary beam with elliptical shape of 6 × 9 µm was used for the measurement of 3 large olivine grains. The primary beam was rastered over an 8 × 8 µm^2^ area during the pre-sputtering for 60 s, and then the raster size was reduced to 1 × 1 µm^2^ for the data collection. ^16^O^−^, ^17^O^−^ and ^18^O^−^ were measured using a multi-collector FC (10^10^ Ω, designated as L′2), an axial FC (10^12^ Ω) and a multi-collector FC (10^12^ Ω, designated as H1), respectively. The secondary ion intensity of ^16^O^−^ was ~1.0 × 10^9^ cps. The data were collected for 40 cycles with 4 s integration time per cycle. Obtained count rates were corrected for FC background, monitored during the pre-sputtering of every measurement, and relative yield of each detector. The ^16^OH^−^ count rate was measured immediately after the measurements, but we did not make a tail correction on ^17^O^−^ because its contribution to ^17^O^−^ was calculated as ~0.002‰. Typical uncertainties for δ^17^O, δ^18^O and Δ^17^O were 0.7‰, 0.5‰ and 0.6‰ (2*σ*), respectively.

An ~30-pA primary beam with elliptical shape of ~1.7 × 2.7 µm (~2.3 × 3.6 µm including beam halo) was used for the measurement of the smaller grains of olivine and pyroxene in Bennu. ^16^O^−^, ^17^O^−^ and ^18^O^−^ were measured using a multi-collector FC (10^11^ Ω, designated as L1), an axial EM and a multi-collector EM (designated as H2), respectively. The secondary ion intensities of ^16^O^−^ were ~1.7–2.6 × 10^7^ cps and ~1.8 × 10^7^ cps for olivine and pyroxene, respectively. The data were collected for 60 cycles with 4 s integration time per cycle. Obtained count rates were corrected for FC background, EM dead time and relative yield of each detector. The ^16^OH^−^ count rate was measured immediately after the measurements, but we did not make a tail correction on ^17^O^−^ because its contribution to ^17^O^−^ was calculated as ~0.02‰. Typical uncertainties for δ^17^O, δ^18^O and Δ^17^O were 1.5‰, 0.9‰ and 1.6‰, respectively.

San Carlos olivine (Mg# = 89; δ^18^O = 5.2‰) and synthetic enstatite^132^ (δ^18^O = 10.55‰) were used as standards to correct the instrumental mass fractionation for olivine and pyroxene, respectively. As the Mg# of olivine grains is >83, variations in instrumental mass fractionations correlated with Mg# of olivine from that of San Carlos olivine^133^ are insignificant considering the analytical uncertainties of this study.

##### The Open University, UK

The samples OREX-501054-0 and OREX-501059-0 were mounted in resin blocks and polished at the NHM, London, during which process the samples fragmented into particles, identified as P1 and P2. Following characterization by SEM/EPMA, an additional carbon coat was added for a total thickness of ~30 nm.

Olivine and pyroxene grains were identified and characterized at the NHM. Major and minor element abundances were acquired using a CAMECA SX100 electron microprobe. Analyses were performed at 20 kV, using a focused 1-μm beam. Typical detection limits for transition metals were around 250 ppm. Additional quantitative data were acquired using a Zeiss EVO 15LS analytical scanning electron microscope with an Oxford Instruments X-Max80 energy dispersive X-ray silicon drift detector. The EDS system was calibrated using an elemental cobalt standard and a Kakanui augite mineral standard at an acceleration voltage of 20 kV and a beam current of 3 nA.

At the OU, oxygen-isotope measurements of 15 grains of olivine and 2 pyroxenes in OREX-501054-0 and OREX-501059-0 were made on the CAMECA NanoSIMS 50L at the OU. The location of each grain was readily identified using the optical system of the NanoSIMS and a 2-pA Cs^+^ beam total ion current imaging of the carbon coat. Analyses were performed with a focused 100-pA Cs^+^ probe (<0.5 µm diameter). Seven secondary ion species were collected simultaneously, with ^16^O^−^ measured on a Faraday detector while ^17^O^−^, ^18^O^−^, ^30^Si^−^, ^26^Mg^16^O^−^, ^42^Ca^16^O^−^ and ^56^Fe^16^O^−^ were measured on EMs. A mass resolving power of ~10,000 (CAMECA definition^122^) was used, which is sufficient to resolve the ^16^OH^−^ interference from the ^17^O^−^ signal. Before analysis, each area was pre-sputtered with a focused 16-kV, 100-pA Cs^+^ probe for 3 min over an area of 4.5 × 4.5 µm. Analyses were performed with a focused 100-pA Cs^+^ probe (<0.5 μm diameter) rastered repeatedly over 2.5 × 2.5 μm in ‘spot’ mode (a 64 × 64 pixel raster lasting 0.54 s). Each analysis, including centring routines, lasted ~7 min, providing a total of ~8 × 10^9^ counts for ^16^O^−^. The ^16^OH^−^ signal was determined at the start and end of each analysis and a tailing correction applied to the ^17^O signal, although in all cases the correction was <0.1‰ apart from one analysis where the correction was 0.4‰.

Olivine analyses were corrected for instrumental mass fractionation against a standard sample of Fo_90_ San Carlos olivine (δ^18^O = 4.91‰, as measured by laser fluorination), and pyroxene samples corrected to a sample of enstatite from the Shallow Water aubrite (SHW-En from ref. ^105^, δ^18^O = 5.69)) that were analysed before and/or after each block of unknown samples. Analytical uncertainty (all 2*σ*), using quadratic combination of internal counting statistics from the sample measurement and external precision from standard replicates analysed before and/or after the samples, is typically ±1.5‰ for δ^17^O, ±1.1‰ for δ^18^O and ±1.0‰ for Δ^17^O. Matrix correction was applied to account for differences in the Fe/Mg of the samples of olivine. As the pyroxene sample composition was close to the pure enstatite standard no additional matrix correction was applied.

The location of each raster pit, as well as absence of any significant cracks or inclusions, was verified using SEM following analyses. Two analyses were discarded because of very irregular sputter pit geometry.

## Supplementary information

Supplementary Information Supplementary Figs. 1 and 2.

Supplementary Data 1 Supplementary Tables 1–14.

## Source data

Source Data Fig. 1 Statistical source data.

Source Data Fig. 2 Statistical source data.

Source Data Fig. 3 Statistical source data.

Source Data Fig. 4 Statistical source data.

Source Data Fig. 5 Statistical source data.

Source Data Extended Data Fig. 1 Statistical source data.

Source Data Extended Data Fig. 2 Statistical source data.

Source Data Extended Data Fig. 3 Statistical source data.

Source Data Extended Data Fig. 4 Statistical source data.

Source Data Extended Data Fig. 6 Statistical source data.

Source Data Extended Data Fig. 7 Statistical source data.

Source Data Extended Data Fig. 9 Statistical source data.

Source Data Extended Data Fig. 10 Statistical source data.

## Data Availability

The instrument data supporting the experimental results in this study are available at https://astromat.org at the DOIs given in Supplementary Table 1 and/or within the article and its Supplementary Information (Supplementary Data 1 contains Supplementary Tables 1–14). Source data are provided with this paper.
