# Supplementary material for: The variety and origin of materials accreted by Bennu’s parent asteroid
Source: Nat Astron. 2025 Aug 22;9(12):1785–802. doi: 10.1038/s41550-025-02631-6 (PMC12708356; doi:10.1038/s41550-025-02631-6)
Supplement: Supplementary file 1 — Supplementary Figs. 1 and 2. [file 41550_2025_2631_MOESM1_ESM.pdf]

nature astronomy

Supplementary information

https://doi.org/10.1038/s41550-025-02631-6

The variety and origin of materials accreted by Bennu's parent asteroid

In the format provided by the authors and unedited

Supplementary Information

The variety and origin of materials accreted by Bennu's parent asteroid

Barnes and Nguyen et al.

Supplementary Figures 1 and 2 support the SIMS and NanoSIMS analyses of anhydrous silicates in the Bennu samples. Supplementary Figure 1 shows the analysis locations of grains analyzed at Hokkaido University, Japan. Supplementary Figure 2 demonstrates the consistency of measured O isotopes in anhydrous silicates between different SIMS and NanoSIMS laboratories.

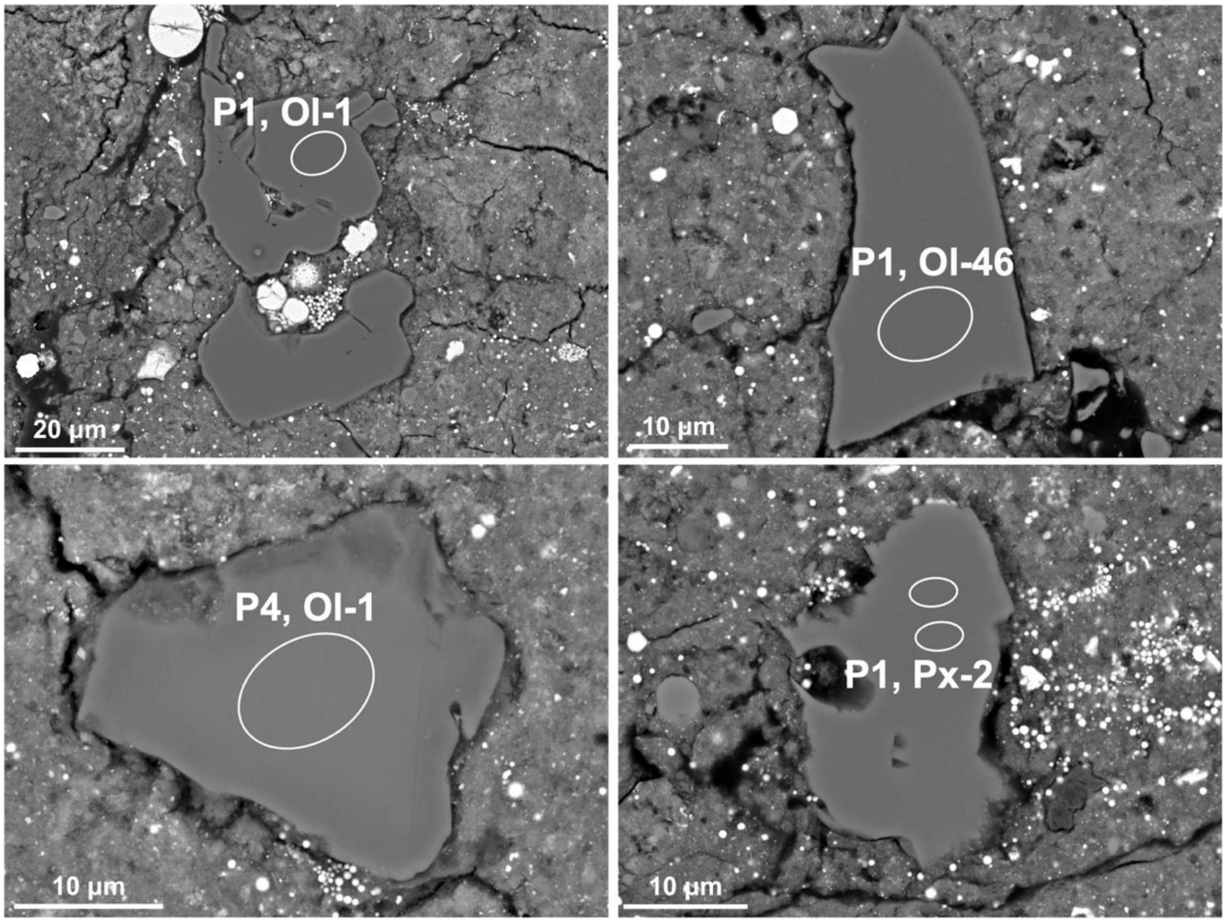

Supplementary Figure 1. Examples of anhydrous silicates studied for O isotopic composition in Bennu sample OREX-803114-0. BSE images of olivine (Ol) and low-calcium pyroxene (Lpx) in Bennu particles. In each case, the anhydrous silicate is surrounded by fine-grained matrix with proximal magnetite framboids and sulfides.

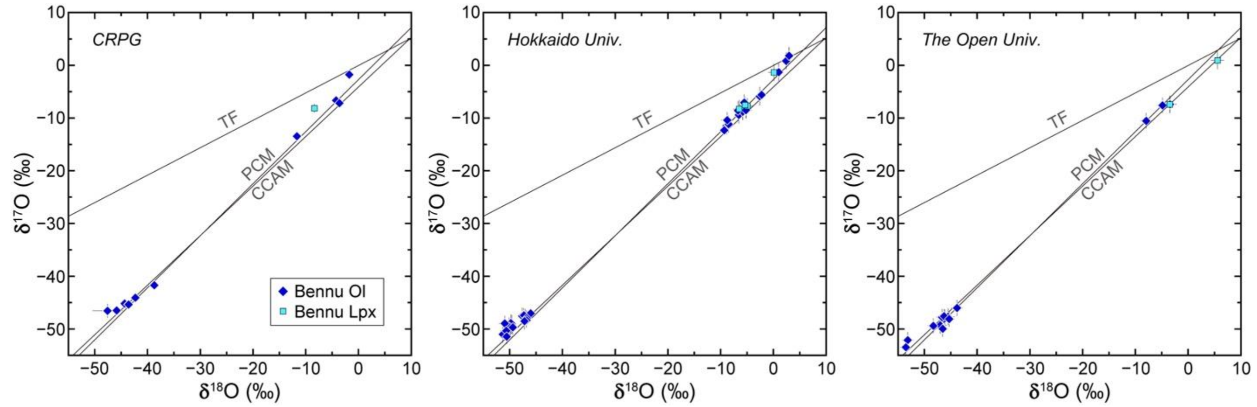

Supplementary Figure 2. Oxygen isotopic compositions of anhydrous silicate minerals in Bennu samples analyzed at Centre de Recherches Pétrographiques et Géochimiques (CRPG), Hokkaido University, and The Open University. Ol = olivine and Lpx = low calcium pyroxene. Data is the same as that presented in Figure 6. Errors are 2SD measurement errors. TF, terrestrial fractionation line; CCAM, carbonaceous chondrite anhydrous mineral line; PCM, primitive chondrule mineral line as presented in the Methods.

Information on the samples studied, the elements and isotopes measured and in which laboratory can be found in Supplementary Table 1. The table also includes the DOIs issues by Astromat for data bundles. Supplementary Tables 2–14, which can be found in the Supplementary Data 1 file, provide the final processed data reported in this study.
